# Gsta4 controls apoptosis of differentiating adult oligodendrocytes during homeostasis and remyelination via the mitochondria-associated Fas-Casp8-Bid-axis

**DOI:** 10.1038/s41467-020-17871-5

**Published:** 2020-08-13

**Authors:** Karl E. Carlström, Keying Zhu, Ewoud Ewing, Inge E. Krabbendam, Robert A. Harris, Ana Mendanha Falcão, Maja Jagodic, Gonçalo Castelo-Branco, Fredrik Piehl

**Affiliations:** 1 Department of Clinical Neurosciences, Karolinska Institutet, Center for Molecular Medicine, Karolinska Hospital at Solna, 17177 Stockholm, Sweden; 2 Laboratory of Molecular Neurobiology, Department Medical Biochemistry and Biophysics, Biomedicum, Karolinska Institutet, 17177 Stockholm, Sweden; 3 Life and Health Sciences Research Institute (ICVS), School of Medicine, University of Minho, Minho, Portugal; 4 Ming Wai Lau Centre for Reparative Medicine, Stockholm node, Karolinska Institutet, Stockholm, Sweden

**Keywords:** Biochemistry, Transferases, Cell death, Post-translational modifications, Multiple sclerosis

## Abstract

Arrest of oligodendrocyte (OL) differentiation and remyelination following myelin damage in multiple sclerosis (MS) is associated with neurodegeneration and clinical worsening. We show that Glutathione S-transferase 4α (Gsta4) is highly expressed during adult OL differentiation and that Gsta4 loss impairs differentiation into myelinating OLs in vitro. In addition, we identify Gsta4 as a target of both dimethyl fumarate, an existing MS therapy, and clemastine fumarate, a candidate remyelinating agent in MS. Overexpression of Gsta4 reduces expression of Fas and activity of the mitochondria-associated Casp8-Bid-axis in adult oligodendrocyte precursor cells, leading to improved OL survival during differentiation. The Gsta4 effect on apoptosis during adult OL differentiation was corroborated in vivo in both lysolecithin-induced demyelination and experimental autoimmune encephalomyelitis models, where Casp8 activity was reduced in Gsta4-overexpressing OLs. Our results identify Gsta4 as an intrinsic regulator of OL differentiation, survival and remyelination, as well as a potential target for future reparative MS therapies.

## Abstract (web summary)

Impaired oligodendrocyte (OL) differentiation and remyelination after myelin damage in multiple sclerosis is associated with neurodegeneration. The authors show that Gsta4 is expressed during adult OL differentiation and identify it as a regulator of OL differentiation, survival, and remyelination.

## Introduction

Oligodendrocyte precursor cells (OPC) and oligodendrocytes (OL) are abundant throughout the central nervous system (CNS). They serve important roles in preserving the functionality and integrity of neuronal network connections, including providing metabolic support and enabling axonal signal transmission along myelinated fibers^1–4^. OLs are affected directly or indirectly in many CNS diseases, of which Multiple Sclerosis (MS) is one of the most widely studied^5^. Despite findings of proliferating OPCs around lesions^6,7^ OLs largely fail to remyelinate axons^8–12^ leading to neurodegeneration^9,13–15^.

Remyelination does occur in adulthood^6,16–19^ but the temporal interval within which the OLs receive crucial support in order to survive is restricted^20–22^. As a consequence, as many as half of OLs undergo programmed cell death during pre-natal development and up to a third during the post-natal phase^23,24^. Recent studies have shed light on some of the underlying mechanisms for OLs loss during development^25,26^, but to what degree these mechanisms are relevant for the mature nervous system is still not known.

We herein addressed if mitochondrial stress and scavenging of detrimental endogenous compounds during OPC differentiation could be one contributing factor for arrest of differentiation^27–30^. Notably, the activity of the primary sensor of cellular stress, nuclear factor (erythroid-derived 2)-like 2 (Nrf2), is diminished with ageing^31,32^ and in MS^33^. As a consequence, mitochondrial function and the capacity to scavenge endogenous toxins are also diminished, with possible impact on disease-processes such as those active in MS^34–36^. Interestingly, capacity of OPCs to remyelinate is also strongly reduced with ageing^37–39^, even if a link with cellular stress has not been proven. However, it is clear that dysfunctional mitochondria and/or toxin scavenging induce lipid peroxidation of unsaturated lipids in membranes for which OLs are particularly sensitive^40,41^ results in interruption of vital intracellular processes. In this study we show that Glutathione S-transferase 4α (Gsta4) is highly expressed during OL differentiation and that *Gsta4* expression is affected by dimethyl fumarate (DMF) and clemastine fumarate through their actions on Nrf2. DMF, a drug widely used to treat MS, has been described to contribute to OL maturation^42^. Clemastine fumarate has shown promising clinical effects in a trial of MS patients with chronic optic neuropathy^40,43,44^, indicating an effect on myelination^41,45^. We report that Gsta4 restricts apoptosis of pre-myelinating OLs via modulation of the mitochondria-associated Fas-Casp8-Bid-axis, thus enhancing the proportion of OPCs that become myelinating OLs. Importantly, Gsta4 also promoted remyelination and ameliorated clinical symptoms of MS-like disease in rodents.

## Results

### DMF and Clem-F upregulate Gsta4 to promote OL maturation

**Fig. 1:**
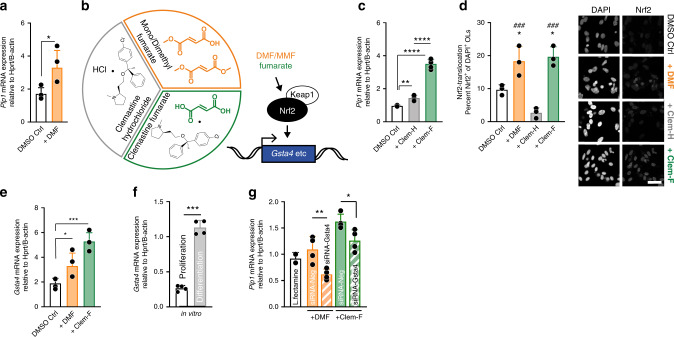
DMF and Clem-F upregulate Gsta4 and promotes OL differentiation. **a** Transcription of *Plp1* in primary OPC cultures following stimulation for 24 h with DMF in DMSO (10 μM) or DMSO control (*n* = 3). **b** Chemical structures of DMF, MMF, Clem-F and Clem-H. **c** OL stimulation with Clem-F and Clem-H (10 μM) for 24 h (*n* = 4). **d** Nuclear activation of Nrf2 following stimulation for 24 h with DMSO Ctrl, DMF, Clem-H, and Clem-F dissolved in DMSO at 10 μM (scale bar 50 μm). Asterisk, comparisons to DMSO control. Number sign, comparison to Clem-H (*n* = 3). **e** Transcription of *Gsta4* in primary OL cultures following stimulation for 48 h with DMF and Clem-F dissolved in DMSO (*n* = 3). **f** Transcription of *Gsta4* in proliferative OPCs compared to differentiated OLs in vitro (*n* = 4). **g** Transcription of *Plp1* in primary OL cultures following stimulation for 24 h with DMF and Clem-F dissolved in DMSO and lipofectamine-mediated knockdown of Gsta4 with siRNA-Gsta4 compared to scrambled sequence, siRNA-Neg (*n* = 4). Graphs show representative results from three (**a**, **c**) or two **d**–**g** independent experiments. All data are shown as mean and S.D. All statistical analyses were performed using one-way ANOVA. *P < 0.05, **P < 0.01, ***P < 0.001, ^###^*P* < 0.001, ****P < 0.0001.

DMF has been described to contribute to OL maturation. Accordingly, we observed that DMF significantly increased mRNA expression of myelin proteolipid protein 1 (*Plp1*) in differentiating neonatal rat OPCs (Fig. 1a, Supplementary Fig. 1b). Clemastine fumarate had promising clinical effects in a trial of MS patients with chronic optic neuropathy^40,43,44^, possibly also influencing remyelination^41,45^. Notably, the clemastine drug formulation used in clinical trials includes a fumarate moiety, thus makes it similar to DMF. In order to elucidate the contribution of the fumarate moiety we evaluated the effect of clemastine fumarate (Clem-F) and clemastine hydrochloride (Clem-H) (Fig. 1b). Both of these increased *Plp1* transcription, but the effect of the latter was significantly greater (Fig. 1c).

The transcription factor Nrf2 has been identified as the main therapeutic target of DMF in several cell types^42,46–48^. Electrophilic agents such as DMF and its active metabolites (e.g monomethyl fumarate (MMF)) facilitates Nrf2 activation via Keap1^49,50^. Indeed we found that DMF and Clem-F caused nuclear activation of Nrf2 in OLs to a larger proportion compared to both control and Clem-H treated OLs (Fig. 1d). Interestingly, Gsta4, the main scavenger of the lipid peroxidation product 4-hydroxynonenal (4-HNE), has been predicted to be a target of Nrf2^51,52^, which we corroborated through the observation that both DMF and Clem-F induced the expression of *Gsta4* (Fig. 1e). DMF also elevated *Gsta4* transcription in brain tissue following oral gavage (Supplementary Fig. 1a). Furthermore, *Gsta4* transcription was strongly increased in OLs during differentiation compared to in proliferating OPCs (Fig. 1f). DMF- and Clem-F-mediated *Plp1* expression was reduced in the presence of Gsta4-specific siRNA (siRNA-Gsta4) knockdown (Fig. 1g, Supplementary Fig. 1c). Gsta4 thus acts downstream of DMF and Clem-F to promote OL differentiation, suggesting an Nrf2-Gsta4-axis operating in the oligodendrocyte lineage.

### Gsta4 leads to expression of genes involved in OL maturation

**Fig. 2:**
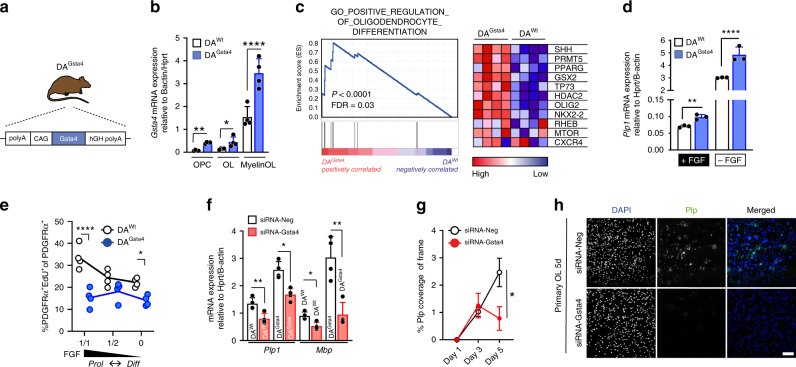
OLs differentiate in a Gsta4-dependent manner. **a** Illustration of Dark Agouti (DA) rat over-expressing Gsta4 (DA^Gsta4^). **b** In vivo transcription of *Gsta4* across the OL lineage in adult DA^Gsta4^ and DA^Wt^ (*n* = 4). **c** GSEA, analyzed using weighted enrichment statistics and ratio of classes for the metric as input parameters, for CNS bulk tissue (cortex, corpus callosum and hippocampus) from adult DA^Gsta4^ and DA^Wt^ (*n* = 4). **d** Transcription of *Plp1* following 48h differentiation of neonatal DA^Gsta4^ or DA^Wt^-derived OPCs in the presence or in absence of bFGF (*n* = 3). **e** In vitro proliferation of DA^Wt^ and DA^Gsta4^ OPCs for 72h in various concentration of Fgf (*n* = 4). **f** Transcription of *Mbp* and *Plp1* following 48 h differentiation of neonatal DA^Gsta4^ or DA^Wt^-derived OPCs in combination with Gsta4 knockdown with siRNA-Gsta4 or siRNA-Neg as a scrambled sequence (*n* = 4). **g** Immunocytochemistry of Plp protein expression following Gsta4 knockdown with siRNA-Gsta4 (*n* = 10). **h** Representative images at day 5, scale bar 50 μm. Graphs depict representative results from two (**b**, **d**–**h**) independent experiments. All data are presented as mean and S.D. All statistical analyses were performed using one-way ANOVA. *P < 0.05, **P < 0.01, ***P < 0.001, ****P < 0.0001.

To study ex vivo effects of Gsta4 in the OL lineage we created a hemizygous rat (DA^Gsta4^) over-expressing Gsta4 under a CAG promotor on a Dark Agouti (DA) background (Fig. 2a). This DA^Gsta4^ strain displayed approximately a two-fold higher levels of Gsta4 mRNA throughout the OL lineage compared to in DA^Wt^, with the highest Gsta4 levels in both DA^Wt^ and DA^Gsta4^ being observed in more mature cells (Fig. 2b). DA^Gsta4^ also displayed elevated *Gsta4* mRNA levels in additional tissues, including brain, spinal cord and spleen (Supplementary Fig. 1g). From a physiological perspective, DA^Gsta4^ did not show obvious phenotypic characteristics in terms of behavior, general health, spontaneous tumors, weight, fertility or litter size as compared to wild type animals (DA^Wt^).

Initial characterization of DA^Gsta4^ was performed using mRNA microarray analysis of bulk tissue from adult (10–12 week old) brain cortex, corpus callosum and hippocampus. Consistent with our in vitro data (Fig. 1g) the transcripts involved in positive regulation inducing OL differentiation were significantly enriched in DA^Gsta4^ compared to DA^Wt^, as assessed by Gene Set Enrichment Analysis (GSEA) (Fig. 2c). To assess if this translated into differences in OL differentiation and maturation markers we determined the expression of *Plp1* and myelin basic protein (*Mbp*) in primary oligodendrocyte cultures established from neonatal rats (Supplementary Fig. 1b). Basic fibroblast growth factor (bFGF) is essential in OPC in vitro expansion, as it promotes proliferation and blocks differentiation (Supplementary Fig. 1b). Upon withdrawal of bFGF, the DA^Wt^ and DA^Gsta4^ OPC pools differentiated. However, DA^Gsta4^ expressed higher levels of *Plp1* both in the presence of bFGF during extended in vitro expansion, and also in its absence upon differentiation (Fig. 2d). Accordingly, there was a lower degree of proliferative (PDGFRα^+^EdU^+^/PDGFRα^+^) DA^Gsta4^ OPCs compared to DA^Wt^ OPCs in both presence or absence of bFGF (Fig. 2e). Nevertheless, while upon short in vitro expansion (Supplementary Fig. 1b) there was a similar decrease of proliferative DA^Gsta4^ OPCs upon differentiation (no bFGF) compared to DA^Wt^, there was slightly higher PDGFRα^+^EdU^+^/PDGFRα^+^ in DA^Gsta4^ compared to DA^Wt^ and decreased *Plp1* when bFGF was present (Supplementary Fig. 1e, f). This suggests that long-term exposure of OPCs to bFGF might prime them to respond by triggering differentiation upon Gsta4 over-expression. To confirm that the effects on DA^Gsta4^ OPC differentiation upon extended in vitro expansion were dependent on Gsta4 we performed knock-down of Gsta4 using siRNAs. Indeed, *Plp1* was dependent on Gsta4 (Fig. 2f). Moreover, the production of Plp protein during differentiation was decreased following addition of siRNA-Gsta4 compared to control siRNA (Fig. 2g, h and Supplementary Fig. 1c). These observations strengthen the notion that Gsta4 regulates differentiation of rat OPCs while exerting context-dependent effects on their proliferation.

### Gsta4 accelerates the passage of OPCs to OLs via Casp8-Bid

**Fig. 3:**
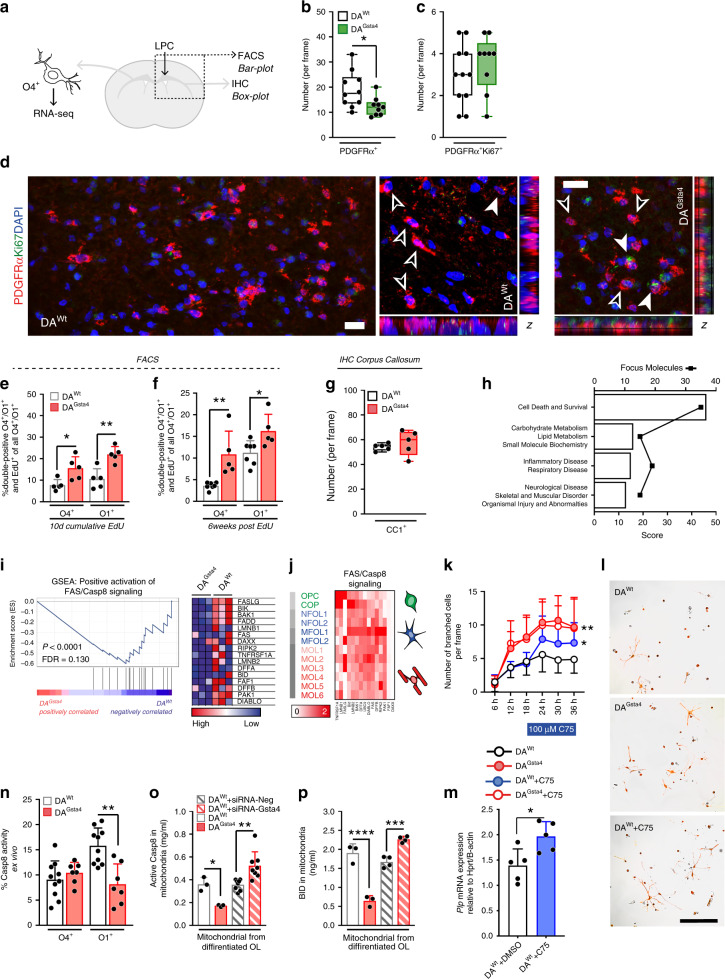
Gsta4 promotes more efficient OL differentiation via Fas-Casp8-Bid. **a** Summary of experimental set-up. **b** Total number of PDGFRα^+^ OPCs per frame in corpus callosum of adult rats (*n* = 10+9). **c** Percentage of double-positive PDGFRα^+^Ki67^+^OPCs of all PDGFRα^+^ OPCs in corpus callosum. **d** Representative images illustrating PDGFRα^+^ (empty arrows) and double-positive Ki67^+^ (filled arrows), scale-bar 10 µm. **e** Percentage of double-positive O4^+^EdU^+^ of all O4^+^ and O1^+^EdU^+^ of all O1^+^ after ten days of EdU administration (*n* = 5) analyzed using flow cytometry. **f** Percentage of double-positive O4^+^EdU^+^ of all O4^+^ and O1^+^EdU^+^ of all O1^+^ 6 weeks following EdU administration (*n* = 7 + 5). **g** Total number of CC1^+^ OLs per frame in corpus callosum (*n* = 5). **h** Top IPA Network from RNA-seq on O4^+^ corpus callosum OLs (*n* = 5). **i** GSEA and heat map of transcripts promoting Fas/Casp8 signaling from RNA-seq on O4^+^ corpus callosum OLs. **j** Heat-map based on transcript levels during OL maturation^20^. **k** Number of branched DA^Wt^ and DA^Gsta4^ OLs per image frame captured with live imaging during 36h of differentiation. The Fas inhibitor C75 was added after 18 h. **l** representative images after 24 h. Overlay with contrast and CellROX staining (red), scale-bar 30 µm. **m** Transcription of Plp1 at 36 h (*n* = 4). **n** Percentage of ex vivo Casp8 activity in O4^+^ and O1^+^ OLs from DA^Wt^ (*n* = 10) and DA^Gsta4^ (*n* = 7). **o** Quantification of active Casp8 derived from mitochondria of DA^Wt^ and DA^Gsta4^ OLs differentiated for 48h, without (*n* = 3) or with (*n* = 8) siRNA. **p** Quantification of Bid derived from mitochondria DA^Wt^ and DA^Gsta4^ OLs differentiated for 48 h, without (*n* = 3) or with (*n* = 4) siRNA. Graphs show representative results from two (**e**, **f**, **k**–**p**) or one (**b**–**d**, **g**) independent experiment(s). All graphs depict mean and S.D., graph **b**–**d** shows box and whiskers indicating values outside 5–95 percentile. All statistical analyses were performed using one-way ANOVA apart from **k**, analyzed with two-way ANOVA. *P < 0.05, **P < 0.01, ***P < 0.001, ****P < 0.0001.

The effects of Gsta4 on the OL lineage were further evaluated in adult DA^Wt^ and DA^Gsta4^ rats as illustrated in Fig. 3a. In line with previous in vitro findings (Fig. 2) adult DA^Gsta4^ had fewer mature OPCs, identified using IHC staining for PDGFRα^+^ (Fig. 3b). Flow cytometric analysis of corpus callosum and grey matter regions (indicated in Fig. 3a, dotted line) corroborated these histological findings evident by decreased numbers of PDGFRα^+^ OPCs and pre-mature O4^+^ OLs in DA^Gsta4^ compared to DA^Wt^ (Supplementary Fig. 1h). Despite a smaller OPC pool in the DA^Gsta4^, a similar number of double-positive PDGFRα^+^Ki67^+^ was observed in the corpus callosum (Fig. 3d and Supplementary Fig. 1i) suggesting a compensatory mechanism for OPC loss, possibly involving bFGF (Supplementary Fig. 1e).

We also analyzed if Gsta4 regulated adult OPC differentiation by assessing EdU levels in O4^+^ and O1^+^ OLs following administration of EdU in drinking water for 10 days and 6 weeks after a single injection of EdU. The proportions of both double-positive O4^+^EdU^+^ and O1^+^EdU^+^ of all O4^+^ or O1^+^ were higher in DA^Gsta4^ at both 10 days and 6 weeks (Fig. 3e, f), suggesting a faster differentiation rate in DA^Gsta4^. However, the total number of CC1^+^ OLs in the adult corpus callosum was not different between DA^Gsta4^ and DA^Wt^ (Fig. 3g and Supplementary Fig. 1h). While a compensation between proliferation and differentiation rates might contribute to the homeostatic levels of OLs in DA^Gsta4^, this finding suggests that other cellular mechanisms might also be involved. To identify pathways contributing to the observed phenotype, O4^+^ OLs were sorted out from micro-dissected adult DA^Wt^ (*n* = 5) and DA^Gsta4^ (*n* = 5) corpus callosum followed by RNA-seq (Fig. 3a). Ingenuity Pathway Analysis (IPA) Network data suggested top networks to be involved in cell survival and death (*Mycn, Egfr, Fas*) as well as carbohydrate and lipid metabolism (*Bicc1, Pisd, Slc25a14*) (Fig. 3h, Supplementary Data 1). GSEA of the RNA from the sorted corpus callosum O4^+^ OLs also revealed lower expression of positive regulators of Fas and Caspase-8 (Casp8) signaling in DA^Gsta4^ (Fig. 3i). A heat-map summarizing the levels of these regulators along the OL lineage also indicated that they were enriched in early myelin forming OLs (MFOL1) (Fig. 3j), indicating a role in the early stages of differentiation/myelination.

In order to address how Fas regulates OL differentiation the Fas antagonist C75 was added to DA^Wt^ and DA^Gsta4^ OPC cultures during differentiation. Under baseline conditions DA^Gsta4^ OLs displayed more branches from the soma as compared to DA^Wt^ OLs. DA^Wt^ branching and *Plp1* expression were significantly increased upon addition of C75 (Fig. 3k–m). Fas signaling thus negatively regulates OL differentiation.

When assessing intracellular mechanisms downstream of Fas by flow cytometry, Casp8 activity ex vivo was indeed lower in O1^+^ DA^Gsta4^ as compared to in DA^Wt^ O1^+^ OLs (Fig. 3n), while no differences were observed in O4^+^ OLs. Casp8 can either activate BH3 interacting-domain death agonist (Bid) or Caspase-3 (Casp3) via catalytic cleavage^53^. Upon cleavage of Bid, both Bid and active Casp8 are associated with the mitochondrial outer membrane^53^. We thus quantified the levels of active Casp8 and Bid in isolated mitochondria from differentiated OLs. We observed a decrease in active Casp8 and mitochondrial levels of Bid in DA^Gsta4^ (Fig. 3o, p), in accordance with the ex vivo findings of reduced Casp8 activity in DA^Gsta4^ OLs (Fig. 3n). In addition, knock-down of Gsta4 in differentiated OLs had the opposite effect, with an increase in mitochondrial levels of Casp8 and Bid (Fig. 3o, p). Taken together this indicates that Gsta4 reduces Fas-Casp8 signaling in differentiating OLs, which might regulate not only their survival but also their differentiation.

### Gsta4 regulates mitochondrial 4-HNE load in OLs

**Fig. 4:**
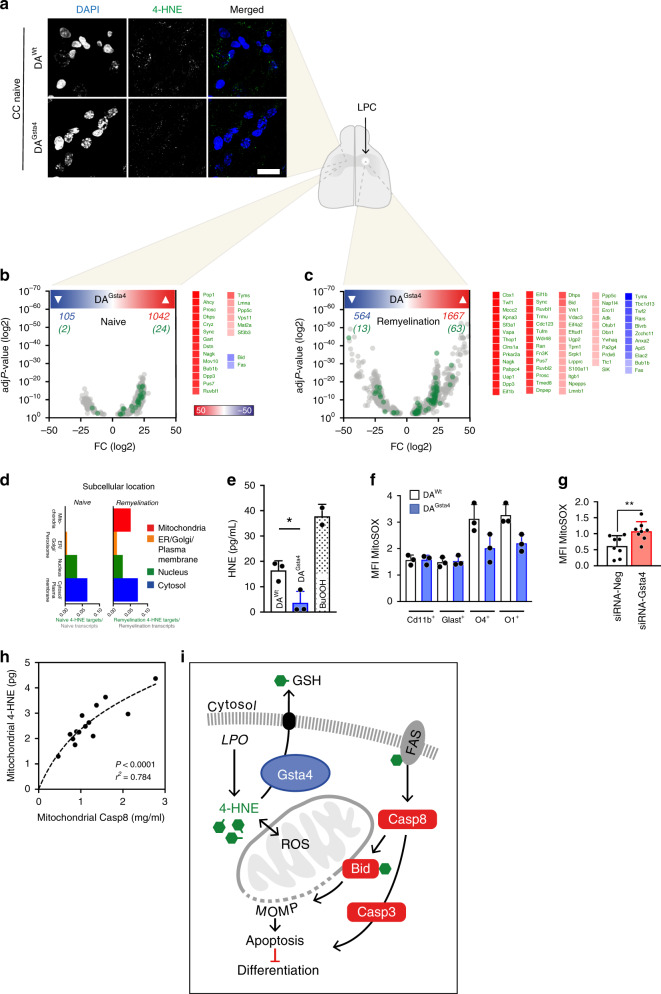
Gsta4 regulates mitochondrial stress and intracellular 4-HNE load in OLs. **a** Staining for 4-HNE in corpus callosum of naïve adult DA^Wt^ and DA^Gsta4^ (*n* = 3), scale-bar 10 µm. **b** Significant transcripts (gray) from RNA-seq of corpus callosum-derived O4^+^ OLs in naïve conditions. Transcripts corresponding to proteins potentially modified by 4-HNE (green)^57,58^. Heat-map indicates up (red) and down (blue) transcripts. **c** Significant transcripts (gray) from RNA-seq of corpus callosum-derived O4^+^ OLs during remyelination. **d** Subcellular distribution of difference in green versus gray transcripts in naïve conditions and during remyelination. **e** Mitochondrial 4-HNE load from differentiated DA^Wt^ and DA^Gsta4^ OLs in culture and BuOOH, inducing LPO, as a positive control (*n* = 3). **f** Flow cytometric assessment of mitochondrial integrity with MitoSOX ex vivo in glial cells and OLs from DA^Wt^ and DA^Gsta4^ (*n* = 2). **g** Flow cytometric assessment of mitochondrial integrity with MitoSOX in differentiated OLs following application of Gsta4 knockdown with siRNA-Gsta4 (*n* = 8). **h** Correlation between mitochondrial 4-HNE load and levels of active Casp8 associated to the mitochondria (*n* = 16). **i** Schematic illustration of Gsta4-mediated 4-HNE transport and possible binding sites for 4-HNE along the Fas-Casp8 pathway. Graphs depict representative results from two (**f**, **h**), three (**e**, **g**) independent experiments. Data in **e**–**g** are shown as mean and S.D. All statistical analyses were performed using two-tailed Student’s *t* test apart from **h** analyzed with two-tailed Pearson’s *r* test. *P < 0.05, **P < 0.01.

4-HNE is the only known substrate for Gsta4 enzymatic activity^54^. In addition, Gsta4 facilitates the extracellular transportation of 4-HNE via conjugation to glutathione (GSH), whereas additional scavenging enzymes show little overlap with Gsta4 function. Intracellular 4-HNE is thus a rate-limiting molecule for several cellular processes, interfering with normal function, protein aggregation and protein degradation^55,56^. We next investigated whether Gsta4 over-expression could work as a model for decrease in intracellular 4-HNE load, resulting in fewer biomolecules being covalently modified by 4-HNE. Indeed, staining for 4-HNE in the corpus callosum revealed the DA^Gsta4^ strain to have a reduced load of 4-HNE compared to DA^Wt^ (Fig. 4a, Supplementary Figure 2a). To identify mechanisms directly affected by Gsta4/4-HNE, we analyzed RNA-seq data of corpus callosum-derived O4^+^ OLs to identify crucial molecules involved in these processes, using the following criteria: (1) significant differential expression (*P* ≤ 0.001) between DA^Wt^ and DA^Gsta4^ (Supplementary Data 2); (2) the potential of the corresponding peptide to be modified by 4-HNE, as analyzed using high performance liquid chromatography screening^57,58^ (Supplementary Data 3). There were 1042 upregulated mRNAs in DA^Gsta4^ OLs compared to in DA^Wt^ in the naïve state, while only 105 mRNAs were downregulated (Fig. 4b left). Of these, 26 of these transcripts encoded proteins that are 4-HNE modified (Fig. 4b green). Transcripts elevated in DA^Gsta4^ included *Ahcy* and *Mat2a*, involved in processes of small carbon units during oxidative stress (Fig. 4b right). As previously observed, *Fas* and *Bid* involved in Casp8-induced apoptosis were downregulated in DA^Gsta4^ in relation to DA^Wt^.

We also assessed involvement of Gsta4/4-HNE in induced demyelination/remyelination, by using the lysolecithin (LPC) model. Specifically, we applied stereotactic administrations of 0.1% LPC into the corpus callosum and sorted O4^+^ OLs during the remyelination phase. Bulk RNA-seq analysis indicated that transcripts such as *Mccc2* and *Vdac3*, involved in mitochondrial gene expression, were differentially regulated between DA^Wt^ and DA^Gsta4^ (Fig. 4c down). Furthermore, the subcellular distributions of transcripts of potentially 4-HNE modified proteins (Fig. 4b, c green) suggested an induction of transcripts coding for proteins localized to the mitochondria during remyelination compared to during naïve conditions (Fig. 4d and Supplementary Fig. 2b).

As mitochondrial stress and subsequent dysfunction is a typical outcome following high intracellular 4-HNE load^30^, we quantified mitochondrial 4-HNE load by isolating mitochondria from OLs differentiated for 48h. DA^Gsta4^ OLs had a reduced mitochondrial 4-HNE load compared to DA^Wt^ and the positive control BuOOH (Fig. 4e and Supplementary Fig. 2c, d), a potent inducer of lipid peroxidation (LPO) generating 4-HNE. Mitochondrial stress was measured using MitoSOX ex vivo in O4^+^ and O1^+^ OLs and in microglia/astrocytes. OLs had a higher MitoSOX signal compared to glial cells but DA^Gsta4^ OLs had a reduced MitoSOX signal compared to DA^Wt^ (Fig. 4f). This was then increased upon addition of siRNA-Gsta4 in vitro (Fig. 4g). Furthermore, mitochondrial 4-HNE load showed a positive correlation with activated Casp8, suggesting that the beneficial effects of Gsta4 on OL apoptosis involves mitochondrial 4-HNE load (Fig. 4h). Taken together our data indicate that Gsta4 over-expression lowers mitochondrial 4-HNE load and downregulates transcripts involved in Casp8-Fas, thereby promoting OL differentiation and survival both during homeostasis and during remyelination (Fig. 4i and Supplementary Fig. 2e).

### Increased remyelination through enhanced OL differentiation

**Fig. 5:**
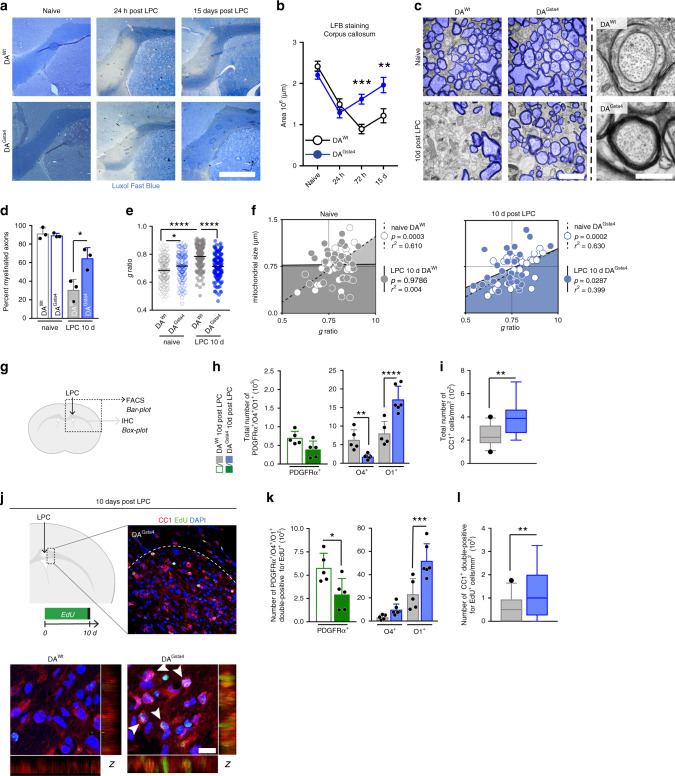
Gsta4 stimulates axonal remyelination through more efficient OL differentiation. **a** Corpus callosum sections stained with LFB for evaluation of demyelination, scale-bar 1 mm. **b** LFB staining following administration of 0.1% LPC (naïve/24h/72h; *n* = 6, 15d; *n* = 5). **c** Representative TEM images of corpus callosum, myelinated axons pseudo-colored in blue, scale-bar 2 μm and 400 nm. **d** Percentage of myelinated axons in naïve DA^Wt^ and DA^Gsta4^ and during remyelination (*n* = 3). **e***g*-ratio in naïve DA^Wt^ and DA^Gsta4^ and during remyelination (*n* = 3). **f** Correlation between *g*-ratio and axonal mitochondrial size during homeostasis (left) and during remyelination (right) (*n* = 30). **g** Illustration of experimental set-up. **h** Flow cytometric analysis of total number of PDGFRα^+^ OPCs (green) (*n* = 5) and O1^+^ and O1^+^ OLs (grey/blue) (*n* = 5 + 6). **i** Immunohistochemically validation of CC1^+^ OLs in corpus callosum (*n* = 5). **j** Representative corpus callosum images 10 days following LPC, scale-bar 10 μm. **k** Number of PDGFRα^+^, O4^+^, or O1^+^ double-positive for EdU^+^ 10 days after continuous EdU administration. **l** Number of CC1^+^ double-positive for EdU^+^ 10 days after EdU administration. All graphs show mean and S.D., apart from **i**, **l** showing box and whiskers, indicating values outside 5–95 percentile. All statistical analyses were performed with one-way ANOVA, apart from **i**, **l** performed with Students’ two-tailed unpaired *t* test and **f** analyzed with two-tailed Pearsons’ *r* test. *P < 0.05, **P < 0.01, ***P < 0.001, ****P < 0.0001.

To address if Gsta4 contributes to repair and remyelination in the context of demyelination, we analyzed DA^Gsta4^ rats upon induction of LPC-mediated demyelination/remyelination (Fig. 5a and Supplementary Fig. 3a). While Luxol fast blue (LFB) staining initially revealed a similar degree of demyelination between the two strains, examination at later time-points revealed a more efficient remyelination in DA^Gsta4^ compared to in DA^Wt^ (Fig. 5b). The difference between strains was already evident at 72 h and persisted until 15 days post injection, a time point when DA^Gsta4^ animals displayed minimal visible demyelinating damage, whereas such damage was still readily evident in the DA^Wt^ strain. No difference in corpus callosum size was evident between DA^Wt^ and DA^Gsta4^ (Supplementary Fig. 3b). Since incomplete scavenging of myelin debris is known to limit OPC differentiation and remyelination, the possible contribution from Gsta4 in microglia during phagocytosis was assessed. pHrodo-labeled myelin was added to primary microglia cultures but no difference in phagocytosis at any time-point between DA^Wt^ and DA^Gsta4^ or siRNA-Neg and siRNA-Gsta4 was observed (Supplementary Fig. 1c, 3c). Moreover, transcription of pro-inflammatory hallmark genes did not vary in bulk corpus callosum tissue at any time-point following LPC exposure (Supplementary Fig. 3d). Neither, was any strain difference in numbers of immune cells or astrocytes following LPC administration were observed (Supplementary Fig. 3e, h–j). Collectively, this indicates that Gsta4 over-expression had no or only a negligible effect on the inflammatory response in the LPC model. To evaluate remyelination of axons, corpus callosum in naïve state and 10 days following LPC injection were evaluated using transmission electron microscopy (TEM) (Fig. 5c and Supplementary Fig. 3f). A two-fold increase in the percentage of myelinated axons was recorded in DA^Gsta4^ compared to DA^Wt^ during remyelination (Fig. 5c, d), where a difference in myelin thickness was also primarily evident in thinner axons (Supplementary Fig. 3f, g). The *g*-ratio describes the diameter of the transected axon in relation to the outer diameter of the surrounding myelin assessed using TEM. Using a software that randomly selects axons for analysis from every image frame^59^, DA^Wt^ axons were determined to be less myelinated after demyelination compared to DA^Gsta4^ axons (Fig. 5c, e and Supplementary Fig. 3f). A small but significant difference was also evident between the strains during homeostasis, as DA^Gsta4^ axons had thinner myelin. We have previously reported that myelin sheath thickness correlates with axonal mitochondria size and that this is an indicator of advanced remyelination^60^, and we herein observed that at day 10 DA^Gsta4^ animals re-established this correlation observed during homeostasis while DA^Wt^ did not (Fig. 5f). DA^Gsta4^ also generated a larger number of O1^+^ and CC1^+^ cells compared to DA^Wt^ 10 days after LPC (Fig. 5g, h and Supplementary Fig. 3h, k). The total number of O4^+^ OLs and PDGFRα^+^ was decreased in DA^Gsta4^ compared to DA^Wt^ following LPC, supporting the notion of a faster remyelinating process in DA^Gsta4^. To validate this, animals were fed EdU in their drinking water for 10 days (Fig. 5j) showing a higher number of DA^Gsta4^ OPCs differentiated into myelin producing O1^+^ OLs or CC1^+^ OLs compared to DA^Wt^ (Fig. 5j–l).

### Gsta4 reduces demyelinated lesions and ameliorates EAE

**Fig. 6:**
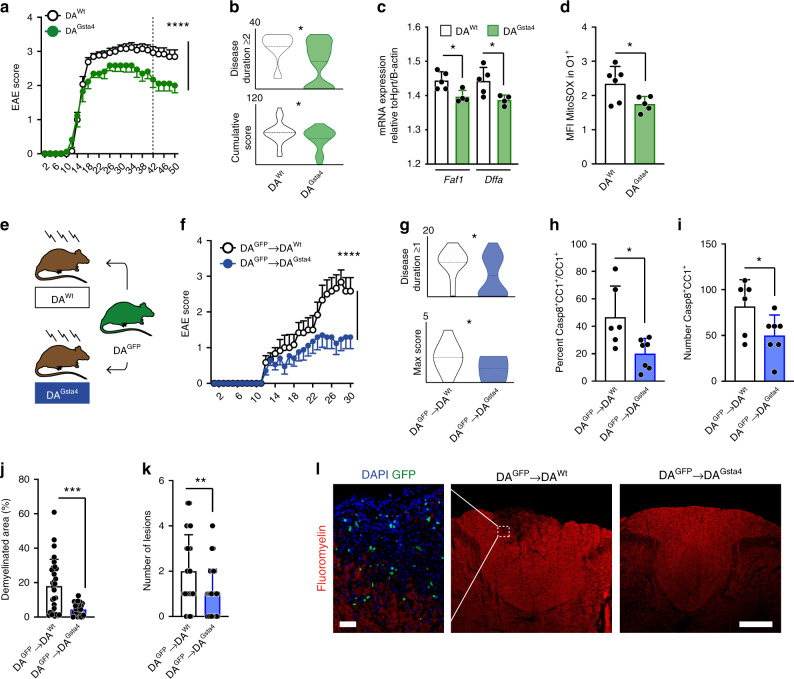
Gsta4 in CNS ameliorates EAE and reduce demyelinating lesions. **a** EAE was induced through subcutaneous injection of MOG in adjuvant (*n* = 26 + 17). **b** Disease duration and cumulative score based on **a**. **c** mRNA expression of Fas-associated *Faf1* and *Dffa* in spinal cord O4^+^ OLs at day 30 (dotted line in **a**). **d** Levels of MitoSOX in O1^+^ OLs from spinal cord at day 50 from **a**. **e**, **f** Illustration of experimental set-up and behavioral EAE score in lethally irradiated DA^Wt^ (*n* = 12) and DA^Gsta4^ (*n* = 16) transplanted with DA^GFP^ bone marrow. **g** Disease duration and max score based on **f**. **h**, **i** Percentage of Casp8^+^CC1^+^ of all CC1^+^ (*n* = 3) and number of Casp8^+^CC1^+^ OLs in spinal cord (*n* = 3) at day 30 based on representative animals in **e**. **j**, **k** Demyelinated area of total area of dorsal column and number of dorsal column lesions (*n* = 3) at day 30 based on representative animals in **e**. **l** Representative images, scale-bar 100 μm. All data are shown as mean and S.D. apart from **b**, **g** showing violin plots and mean. Two-way ANOVA test was used for **a**, **f** to analyze differences between groups over-time, graphs are showing mean and S.E.M. Remaining statistical analyses were performed using Student’s two-tailed unpaired *t* test, apart from disease duration and number of lesions analyzed with Mann–Whitney test. *P < 0.05, **P < 0.01, ***P < 0.001, ****P < 0.0001.

To address the implications of Gsta4 over-expression in a model of autoimmune demyelination we induced experimental autoimmune encephalomyelitis (EAE) in DA^Wt^ and DA^Gsta4^ rats by sub-cutaneous injection of recombinant myelin oligodendrocyte glycoprotein (MOG) in adjuvant. There were no differences in terms of disease incidence or day-of-onset between the groups (Supplementary Fig. 4a), suggesting that the triggering of an autoimmune response did not differ between the strains. However, after the onset of disease animals over-expressing Gsta4 displayed a milder disease course (Fig. 6a), with reduced disease duration and cumulative disability scores compared to DA^Wt^ (Fig. 6b). As observed in corpus callosum O4^+^ OLs during homeostasis (Fig. 3i), mRNAs of mediators partaking in the Fas-Casp8-Bid-axis were also differentially regulated in the spinal cord O4^+^ OLs comparing DA^Gsta4^ and DA^Wt^ at day 42 after immunization (Fig. 6c). In line with this, at the end of experimental follow up, the MitoSOX signal in O1^+^ was lower in DA^Gsta4^ compared to in DA^Wt^ (Fig. 6e and Supplementary Fig. 4b). We could not observe any differences in the numbers of infiltrating leukocytes or activated microglia in the spinal cords between DA^Gsta4^ and DA^Wt^ (Supplementary Fig. 4c), suggesting that Gsta4 did not affect either adaptive or innate immunity.

In order to further substantiate this notion we repeated EAE experiments in bone marrow transplanted DA^Wt^ and DA^Gsta4^ (Supplementary Fig. 4d, e). Animals were lethally irradiated without any shielding and transplanted with green fluorescent protein (GFP)-expressing bone marrow cells from DA^GFP^, followed by immunization with MOG in adjuvant (Fig. 6e). DA^Gsta4^ animals transplanted with DA^GFP^ bone marrow (DA^GFP^→DA^Gsta4^) recapitulated the EAE phenotype previously observed in DA^Gsta4^ (Fig. 6f). Disease duration and maximal disease scores were significantly lower in DA^GFP^→DA^Gsta4^ compared to in DA^GFP^→DA^Wt^ (Fig. 6g). In line with decreased mRNA transcription of Fas-Casp8-Bid-axis mediators and decreased mitochondrial stress in DA^Gsta4^ compared to DA^Wt^, DA^GFP^→DA^Gsta4^ showed lower levels of active Casp8 staining in CC1^+^ spinal cord (Fig. 6h, i). This together rendered in a smaller area of demyelination and fewer lesions in the spinal cord dorsal column in DA^Gsta4^ compared to DA^Wt^ (Fig. 6j–l). Taken together, this set of experiments suggests that Gsta4 over-expression contributes to a lowering of mitochondrial stress and activation of Casp8, which in turn results in less extensive demyelination and/or improved remyelination, as well as ameliorated clinical symptoms.

## Discussion

There has been some progress in the identification of signaling pathways involved in OL maturation. These include the Wnt pathway, interfering with remyelination in the mammalian CNS^61^ and retinoid acid receptor RXR-γ signaling that enhances OPC differentiation and remyelination^62^. A large scale high-throughput screening of compounds with remyelinating effects identified a cluster of anti-muscarinic formulations, including clemastine, that enhance OPC differentiation and remyelination in vitro^43^. Clemastine is a first generation anti-muscarinic compound has been identified as a promising candidate for remyelinating therapy in clinical settings^40,43,44^. We herein demonstrate that both Clem-F and DMF promote OL differentiation in a Gsta4-dependent manner and that this involves Nrf2. The anti-muscarinic formulation contains fumarate, which represents the bioactive molecule following DMF metabolism. This was the basis for addressing the possible overlapping features between Clem-F and DMF to activate Nrf2 in OLs. We show that Clem-F activates Nrf2 and also Gsta4 to similar extent as does DMF, likely via the fumarate moiety. Importantly, we also observed a significant reduction in OL differentiation following DMF and Clem-F application upon siRNA against Gsta4, which illustrates the importance of scavenging enzymes and cellular protection during differentiation. Collectively, our findings suggest that the myelination-promoting effects of both DMF and Clem-F involve the Gsta4/4-HNE pathway and that Gsta4 is essential for mediating their beneficial effect on myelination. Further studies are needed to explore the role of Gsta4 in a clinical context and if manipulation of Gsta4 activity can be used to improve therapeutic outcomes.

Despite detailed knowledge of the molecular heterogeneity between juvenile and adult OL maturation^63^, there are still large gaps in our understanding of what prevents OLs from remyelinating a damaged adult CNS. Our findings suggest that newly formed/myelin-forming OLs represent a particularly sensitive state during adult OL differentiation. This may represent an important rate-limiting step during maturation that could benefit from enhanced Gsta4-activity.

Our approach to identify pathways directly affected by Gsta4 over-expression and sequent reduction of mitochondrial 4-HNE load consisted in overlapping two independent databases of 4-HNE modified proteins, together with assessing their transcription in the OL lineage. The transcripts for *Fas* and *Bid* were the only significantly downregulated in DA^Gsta4^ OLs compared to DA^Wt^. Both Fas and Bid protein have been described to be 4-HNE-modified. Fas can be modified by 4-HNE at three different sites, including one modification within its active site. We confirmed that lowering of Fas (activity) was beneficial to OLs since addition of Fas inhibitor C75 led to an increased differentiation in DA^Wt^ but not in DA^Gsta4^ OLs. The relevance of this pathway was further underlined by reduced activity of Casp8 in DA^Gsta4^ OLs ex vivo. Both mitochondrial Casp8 and Bid were negatively correlated with Gsta4 expression, further proving mitochondria as a relevant location for the intersection between 4-HNE and additional pathways, including the Fas-Casp8-Bid-axis. We thus speculate that the decline in 4-HNE load could be of importance for specific maturation checkpoints during OL differentiation and enables more efficient intracellular processes generally.

Interestingly, Gsta4 also affected OPC proliferation both in cultures derived from neonatal rats in vitro and adult animals in vivo. This effect appears to be context-dependent and may also involve compensatory mechanism to tackle the reduction of OPCs, possibly involving bFGF.

Collectively, our findings suggest that Gsta4 and physiological levels of 4-HNE regulate differentiation of OLs and that this has clinical relevance in in vivo models of inflammation- or toxin-induced demyelination. Tracking of maturing OLs by EdU incorporation after focal demyelination supports the notion that DA^Gsta4^ OLs more rapidly advance through intermediate states of differentiation to more rapidly make up a higher number of myelinating cells in order to enclose damaged corpus callosum axons. The corpus callosum of DA^Gsta4^ is nearly completely restored 15 days following focal demyelination whereas DA^Wt^ still have tangible lesions. This was also supported by TEM analysis in which we observed that DA^Gsta4^ had double the number of myelinated axons 10 days following demyelination. This is despite a similar initial demyelination in both strains. Interestingly, DA^Gsta4^ axons in naïve adult animals appeared to have a slightly thinner myelin sheet. The OPC pool is also reduced in DA^Gsta4^, an explanation for both of these observations possibly being that upon excess of extracellular 4-HNE, transportation cellular processes will operate more efficiently. Thus a smaller pool of precursor cells or thinner myelin is then sufficient to fulfill the function of metabolic support and axonal transmission^60^.

Finally, we observed that Gsta4 over-expression leads to behavioral amelioration upon induction of MS-like disease (EAE). Several reports have suggested differences between species regarding adult remyelination^18,64,65,66^ or that precisely timed input is needed^67^, however EAE is a widely accepted model for MS-like disease in rodents. Gsta4 did not affect time of onset or incidence but improved disease duration and cumulative and maximum disability scores, also upon depletion of Gsta4 over-expression from the immune system. This could further be linked to mitochondrial dysfunction and Casp8, which were both reduced in DA^Gsta4^ OLs. EAE in non-chimeric animals displayed a difference in mortality, since this was not present in the chimeric EAE set-ups, so one cannot exclude Gsta4-dependent contributions from bone marrow-derived cells in non-chimeric EAE setting.

Facilitating and increasing remyelination has been a longstanding therapeutic goal in chronic demyelinating diseases such as MS^68^. Until very recently successful examples, especially in clinical settings, have been lacking. Our study places Gsta4 as a key regulator of OL differentiation and remyelination in the context of cellular stress, ageing and demyelination in an experimental setting.

## Methods

### Study design

Our research object was to investigate the possible beneficial role of Gsta4, activated by DMF and Clem-F, in remyelination and in vivo OL differentiation in MS disease-like models. The DA^Gsta4^ rat was thus designed and used to over-express Gsta4 both in primary cultures and in in vivo models in which siRNA towards Gsta4 was used as a knock-down tool. All primary OL cultures were established from neonatal rats (3–7 days) (Supplementary Fig. 1b) whereas adult animals at an age of 8–10 weeks were used for bone marrow transplantation and later EAE after an additional 8–10 weeks. For remaining experiments adult animals at an age of 10–12 weeks were used. Throughout this study littermate controls were used and all cages contained animals with different genotypes/treatment. OL differentiation was evaluated in the corpus callosum using PDGFRα^+^ to label OPC and CC1^+^ to label post-OPCs. Upon handling and evaluation with IHC and LFB the investigator was unaware of the genotype of the animals/sections.

### Animals and genotyping

The rat DA^Gsta4^ strain over-expressing *Gsta4* under a CAG-promotor was purchased from Taconic Bioscience. DA^GFP^ animals were kindly provided by Holger Reichardt’s laboratory. Animals were genotyped using PCR and vector-specific primers (F: ATCCACTTTGCCTTTCGCGCC, R: TTTCAAACACTGGGAAGTAACG), and primers toward Cd79b (F: GACTCTGTGGCTGCTCATCC, R: TTCAGCAAGAGCTGGGGAC) as wild-type allele control. Animals were bred in the animal facility at Karolinska University Hospital (Stockholm, Sweden) in a pathogen free and climate-controlled environment with regulated 12 h light/dark cycles. All experiments were approved and performed in accordance with Swedish National Board of Laboratory Animals and the European Community Council Directive (86/609/EEC) under the permits N275-15 and N244-13.

### Bone marrow transplantation and EAE

DA^GFP^ male animals were sacrificed with carbon dioxide and femurs was removed and rinsed with cold PBS in order to harvest hematopoietic stem cells (HSC). These were washed, passed through a 40μm strainer and erythrocytes were lysed using ACK buffer (Thermo Fisher Scientific, Waltham, MA) following the manufacturer´s protocol. At an age of 8–10 weeks, male BM recipients were subjected to lethal irradiation with 2 × 5Gy and subsequently given 10 × 10^6^cells/in 300 μL PBS i.v. Successful transfer of the bone marrow graft was assessed by analyzing GFP^+^ cells in the peripheral blood 8 weeks after transplantation using flow cytometry. EAE in bone marrow transplanted male animals was induced in anesthetized 8–10-week-old animals after irradiation by subcutaneous injection at the tail base of 10 μg of recombinant rat MOG together with incomplete Freud’s adjuvant 1:1 (Sigma, St Louis, MO). EAE was induced in 10–12-weeks-old female animals using the same protocol using 10 μg MOG. The animals were monitored daily from onset of disease and scored according to the following scheme: 0 = no clinical score, 1 = reduced tail tonus, 2 = hind leg paraparesis or hemiparesis, 3 = hind leg paralysis or hemiparalysis, 4 = tetraplegia or moribund, 5 = death. Rats were scrificed if they showed severe balance disturbance or abnormal behavior, >25% weight loss or prolonged tetraplegia. Removed animals were scored 4 throughout (Supplementary Fig. 4a). EAE experiments were repeated twice (EAE1 *n* = 17 + 26; EAE2 *n* = 16 + 18) and twice in bone marrow transplanted animals (BM-EAE1 *n* = 12 + 16; BM EAE2 *n* = 10 + 14) with comparable and significant results.

### Lysolecithin injections

Male rats animals 10–12 weeks were anaesthetized with isoflurane and subjected to a stereotactic injection into the corpus callosum (AP: 1.0 mm; L:1.2; DV: 2.2) (Supplementary Fig. 3a) with 2.5 μL 0.1% lysolecithin (Sigma, St Louis, MO) in PBS over 5 min using a Hamilton syringe 1710RN (Sigma, St Louis, MO)^69,70^. Animals were kept for 24 h, 72 h, 10 day or 15 day and evaluated with flow cytometry, qPCR, TEM or immunohistochemistry. For in vivo differentiation, 5-ethynyl-2′-deoxyuridine (EdU; Thermo Fisher Scientific, Waltham, MA) was either injected i.p. 25 mg/kg, or added to the drinking water (0.2 mg/mL) for a period of 10 day following LPC injection or in naïve animals for the same time period. Injected animals were kept for 6 weeks.

### Primary OPC cell cultures

For rat primary OPC cell culture preparation brains were collected from neonatal rats (3–7 day). The tissue was incubated in 1 mL Accutase and 4 μL DNase/mL (Sigma, St Louis, MO) at +37 °C and passed through a fire–polished Pasteur pipette every 10 min, this being repeated three times with decreasing pipette guage. The homogenate was directly labeled and isolated using magnetic anti-A2B5 beads (130-093-388) (Milteny Biotec, Bergisch Gladbach, Germany) instead of Percoll layering as for flow cytometry. Throughout the study, culture ware was pre-coated with Poly-l-lysine (Sigma, St Louis, MO) at +37 °C for >2 h, rinsed with distilled water twice and incubated with Fibronectin (Sigma, St Louis, MO) at +37 °C for >1 h. Cells were cultured in 200 μL Neurobrew (Milteny Biotec, Bergisch Gladbach, Germany), 100 μL N2-supplement (Thermo Fisher Scientific, Waltham, MA), 2 μL bFGF (PeproTech, Princeton, NJ) and 2 μL PDGF-BB (R&D Systems, Minneapolis, MA) per 10 mL of DMEM/F-12 media (Thermo Fisher Scientific, Waltham, MA). For differentiation bFGF and PDGF-BB was removed from the media. Cells were proliferated for 5–7 day at +37 °C and 5% CO_2_ in a humidified incubator, harvested with Accutase and re-seeded; 50 000cells/well for 96-well plate (Nunc, Rochester, NY) or 75-100 000cells/well for staining in chambers (177402PK) (Nunc, Rochester, NY).

### siRNA targeted knockdown

For siRNA knockdown samples were transfected with 1 pmol siRNA together with 0.3 μL Lipofectamine (Thermo Fisher Scientific, Waltham, MA) per 200 μL and incubated for 24 h in media. siRNA sequences were design using Thermo Fisher online software, siRNA-Gsta4 (#15) (used throughout): sense; GCAUUUAAGACAAGAAUCAtt; antisense; UGAUUCUUGUCUUAAAUGCct, siRNA-Gsta4 (#22) (only used in Supplementary Fig. 1): sense; GGAUGGAUGCCUGCUUUUUtt, antisense; AAAAAGCAGGCAUCCAUCCtt, siRNA-Neg (#4404021) (Thermo Fisher Scientific, Waltham, MA).

### OL stimulation with DMF, Clem-F, Clem-H, and C75

DMF (PubChem ID: 637568) and Clem-F (PubChem ID: 5281069) (Sigma, St Louis, MO) and Clem-H (PubChem ID: 66847663) (BOC Science, Shirley, NY) were dissolved in DMSO and added to differentiation media at a final concentration of 10 μM 24 h. An equal amount of DMSO was added to control samples. OL differentiation was quantified with RT-qPCR and anti-Plp staining, described below. The Fas inhibitor C75 (Sigma, St Louis, MO) was dissolved in DMSO and added to differentiation media at a final concentration of 100 μM for 18 h following 18 h of differentiation. An equal amount of DMSO was added to control samples. When switching to differentiation media OLs were labeled with CellROX Deep Red (Thermo Fisher Scientific, Waltham, MA) at a final concentration or 1000 nM. OL differentiation was quantified with live cell imaging using IncuCyte^TM^ ZOOM (Essens Bioscience, Göttingen, Germany). Deep red signal and contrast were acquired every second hour for 36 h. The number of branched cells was used as a measurement for OLs differentiated cells.

### Immunocytochemistry

For immunocytochemistry cells were washed and fixed in 100 μL 4% PFA at +4 °C for 10 min, washed with PBS and incubated with 0.1% Triton X-100 for 5 min at +4 °C. For Plp-staining cells were washed and stained with anti-Plp 1:500 (NB100-1608) (Novous Biologicals, Littleton, CO) overnight followed by anti-chicken-Cy3 (Thermo Fisher Scientific, Waltham, MA) and co-stained with CellMask and Hoechst (Thermo Fisher Scientific, Waltham, MA) for 5 min at +4 °C. Co-localization was also performed due to the manufacturer’s protocols using Puromycin (Sigma, St Louis, MO) as a negative control and anti-HNE (ab48506) (Abcam, Cambridge, GB) (1:50) and anti-Pus7 (PA5-54983) (Thermo Fisher Scientific, Waltham, MA) (1:200). Confocal images were for above described applications were acquired with a DMI6000 microscope (Leica Biosystems, Wetzlar, Germany) and analyzed using ImageJ default plugins for co-localization and staining intensity.

### OPC proliferation

Primary cultures of OPCs derived from neonatal rats were expanded and reseeded 500,000cells/well in a 48-well plate. Settled cells were cultured with 10 µM EdU for 72h in media containing Neurobrew, N2 and PDGF-BB as described above. In addition, media was supplemented with full (1/1), half (1/2) of absent (0) of bFGF concentration referred to the concentration described above. Cells were harvested with Accutase, labeled with anti-PDGFRα (Milteny Biotec, Bergisch, Germany). Dead cells were excluded using near IR Live/Dead (Thermo Fisher Scientific, Waltham, MA). Samples were analyzed with a 3-laser Beckman Coulter Gallios using Kaluza Software (Beckman Coulter, Brea, CA).

### Microglia phagocytosis

For primary microglial cultures brains were collected from rats 10–12 weeks as described above. The cell pellet obtained after Percoll layering was plated in non-precoated culture ware for adherent cells and expanded for 4 days in DMEM/F-12 media with 10% FCS (Sigma, St Louis, MO). Microglia were isolated using anti-Cd11b beads (130-049-601) (Milteny Biotec, Bergisch Gladbach, Germany) due to manufacturer’s exact instructions. pHrodo-labeled rat-myelin was kindly provided by Rasmus Berglund, Karolinska Institutet, and was added to the media for 3h and 6h followed by flow cytometric analysis as described above using anti-CD11b (WT5. ROU) (BD, Franklin Lakes, NJ).

### Flow cytometry and Caspase-8 activity

Animals were sacrificed with carbon dioxide and perfused with PBS, the brain was removed and minced using a sterile scalpel. Tissues were processed as described for primary OPC cultures. Following enzymatic digestion, the homogenate was filtered through a 70 μm strainer and myelin was removed by a 37% Percoll layer spun at 800 × *g* for 10 min at +10 °C without any brake. The pellet was re-suspended to a single cell suspension and stained for EdU Click-IT (Thermo Fisher Scientific, Waltham, MA) according to manufacturer’s instructions and then co-stained with anti-PDGFRα (APA5) (Novous Biologicals, Littleton, CO), anti-O4 (130-118-978) (Milteny Biotec, Bergisch, Germany), anti-O1 (FAB1327V) (Novous Biologicals, Littleton, CO) and anti-CD45 (OX-1 (ROU)) (BD, Franklin Lakes, NJ). Dead cells were excluded using near IR Live/Dead (Thermo Fisher Scientific, Waltham, MA). Caspese-8 activity was measured by ex vivo incubation of single cell suspension, prior to Ab-staining described above. The single cell suspension was incubated for 1h at 37 °C in 300 uL DMEM/F12 media, supplemented with N2 and Neurobrew. FAM-LETD-FMK Caspase 8 reagent (Thermo Fisher Scientific, Waltham, MA) was added at the concentration described by the manufacturer and stained. Samples were analyzed using a 3-laser Beckman Coulter Gallios using Kaluza Software (Beckman Coulter, Brea, CA).

### Cell sorting, microarray, and RNA-seq

Animals were sacrificed and perfused as described above, corpus callosum was dissected and prepared as samples for flow cytometry. The single cell suspension was labeled with magnetic anti-CD11b (130-049-601) and anti-Glast (130-095-825) beads at a dilution suggested by the manufacturer and incubated and separated using LS columns. The negative fraction was stained with anti-O4 beads (130-094-543) and isolated using MS columns. Beads, reagents and columns were provided by Milteny Biotec, Bergisch Gladbach, Germany. RNA was isolated using RNeasy Micro Kit (Qiagen, Venlo, Netherlands) according to the manufacturer´s instructions with minor modifications to the protocol. In brief, isolated cells were lyzed in 500 μL Qiazol following sorting, 100 μL chloroform were added and samples spun at 2500 × *g* for 15 min at +4 °C in a MaXtract column (Qiagen, Venlo, Netherlands). Mixed with 500 μL 70% EtOH and spun at 3000×*g* for 30 s at +4 °C in a MiniElute column, washed with 350 μL RW1, on-column digestion for 15 min at room temperature, washed with 350 μL RW1, 500 μL RLT, followed by 500 μL 80% EtOH and spun for 2 min. RNA was eluted using 10 mM Tris pH7.5. Buffer. Next generation sequencing and bioinformatics analysis was performed by the National Genomics Infrastructure (NGI) at the Science for Life Laboratory on the HiSeq 2500 System using the HiSeq Rapid SBS Kit v2 (Illumina, San Diego, CA) generating >13.5 M reads/sample. Reads were mapped to the Rnor_6.0 reference genome using STAR (Dobin et al 2016). Data normalization and analysis of differential gene expression was done using the DESeq2 R-package (Love et al 2014) using a negative binomial test. The false discovery rate-adjusted P-value (FDR) was estimated based on Benjamin-Hochberg correction. For microarray and qPCR analysis of bulk tissue from naïve DA^Gsta4^ (*n* = 4) and DA^Wt^ (*n* = 4) animals, including cortex, hippocampus and corpus callosum was prepared as the sorted samples using a tissue raptor (Qiagen, Venlo, Netherlands) for lyzing in Qiazol. Sub sequent steps were performed similar to RNA extraction form sorted cells described above. Samples were analyzed using a RaGene-2_1.st array (Affymetrix, Santa Clara, CA) by the Array and Analysis facility at Uppsala University. GSEA was performed using http://www.genomespace.org and gene set GO_POSITIVE REGULATION_OF_OLIGODENDROCYTE_DIFFERENTIATION and were calculated by GSEA with weighted enrichment statistics and ratio of classes for the metric as input parameters. Prediction of subcellular locations was generated based on Supplementary Data 2 and 3 using www.subcellbarcode.com.

### Histopathological analyses and immunohistochemistry

Animals were sacrificed with carbon dioxide and perfused with PBS followed by 4% formaldehyde and post-fixed in formaldehyde for 24 h followed by 3 d in 40% sucrose, snap frozen in iso-pentane and kept at −80 °C. Sections (12 μm) were prepared in a cryostat and kept at −20 °C. The EdU staining (Thermo Fisher Scientific, Waltham, MA) was done according to manufacturer´s instructions. For additional staining’s, sections were blocked in 3% normal goat serum (Sigma, St Louis, MO) for 1 h, repeatedly washed with PBS and incubated with primary antibodies; anti-PDGFRα (ab5460) (1:100), anti-CC1 (OP80) (1:100) (Millipore, Darmstadt, Germany), anti-Casp8 (1:200) (NB100-56116) (Novous Biologicals, Littleton, CO) or anti-HNE (1:50) (ab48506) (Abcam, Cambridge, GB) overnight. All secondary antibodies were produced in goat and labeled with either Alexa Fluor 488, 594, Cy3 or Cy5 (Thermo Fisher Scientific, Waltham, MA). Following PBS wash secondary Abs were incubated 1:400 for 1 h at room temperature. Sections were again washed and mounted with medium containing DAPI (Sigma, St Louis, MO). For LFB (Sigma, St Louis, MO), sections were dried for 30 min, hydrated in 95% EtOH for 2 min, incubated 2 h in LFB at +60 °C, washed in dH_2_O, then washed in 95% EtOH, dH_2_O, freshly prepared 0.05% Li_2_CO_3_, 70% EtOH and washed in H_2_O. Sections were analyzed using a Nikon Eclipse E600 microscope and scanned using a Nikon LS-2000 film scanner (Nikon, Minato, Japan) The LFB staining was analyzed using ImageJ software by measuring corpus callosum size and LFB intensity in black/white transposed images.

### Transmission electron microscopy and analysis

For Transmission electron microscopy (TEM) evaluation of corpus callosum rats were perfused as described above using 2.5% glutaraldehyde, 1% paraformaldehyde in 0.1M PBS. Rinsed with 0.1PBS and post-fixed in 2% OsO_4_ in 0.1M PBS at +4 °C for 2 h. Brains were dehydrated in 70%-OH for 30 min +4 °C, 95%-OH for 30 min +4 °C, 100%-OH 20 min RT, Acetone 2x15min RT, LX-112/Acetone (1:2) 4h RT, LX-112/Acetone 1:1 overnight RT, LX-112/Acetone (2:1), overnight RT, LX-112 overnight RT. Embedding in LX-112 at +60 °C. Embedding and sectioning were performed and pictures taken by EMiL, Clinical Research Center, Department of Laboratory Medicine, Karolinska Institutet, Huddinge, Sweden. Approximately 150 axons from three animals per condition were sub-sequentially analyzed randomly and blinded using ImageJ and *G*-ratio free plugin^59^.

### Mitochondria isolation and ELISA

Mitochondria was isolated from primary OL cultures using Mitochondria Isolation Kit for Cultured Cells (#89874) (Thermo Fisher Scientific, Waltham, MA) as per the manufacturer’s exact instructions. The lysed mitochondria were kept at −80 °C until analysis and diluted 1:1 in sample diluent. Proteins from whole primary OL cultures were obtain by lysing cells in RIPA buffer (Thermo Fisher Scientific, Waltham, MA) and sub subsequential sonication after removal of media and repeated washes with PBS. The lysed cells were kept at −80 °C until analysis and diluted 1:1 in sample diluent. α-Tocopherol (50 µM) and tert-Butyl hydroperoxide (25 µM) (Sigma, St Louis, MO) were used as negative and positive controls and added for 2h to the cultures. The Casp8 ELISA (EKR1606) (Nordic Biosite, Sweden), Bid ELISA (NBP2-69968) (Novous Biologicals, Littleton, CO) and the competitive ELISA for detection of 4-HNE (CSB-EQ027232RA) (Cusabio Technology, Huston, TX) was performed due to the manufacturers exact instruction.

### Quantitative real-time PCR

Total RNA was isolated from tissue or cells using Qiazol and RNeasy mini kit (Qiagen, Venlo, Netherlands) according to manufacturer´s instructions and with 15 min on-column DNase digestion (Qiagen, Venlo, Netherlands). cDNA was prepared with reverse transcriptase using iScript kit (BioRad Laboratories, Hercules, CA). Amplifications were conducted using Bio-Rad SYBR green according to manufacturer’s instructions and plates were run in Bio-Rad CFX optical system (BioRad Laboratories, Hercules, CA). Primers were design to work at +60 °C and to span an exon junction using online software at www.ncbi.nlm.nih.gov. Primer specificity was assessed by determining amplicon size using gel electrophoresis and melt curve analysis of each reaction indicating a single peak. *Bactin* F: CGTGAAAAGATGACCCAGATCA; R: AGAGGCATACAGGGACAACACA, *Hprt*: F: CTCATGGACTGATTATGGACA; R: GCAGGTCAGCAAAGAACTTAT, *Gsta4* F:CAGGAGTCATGGAAGTCAAAC; R: TTCTCATATTGTTCTCTCGTCTC, *Plp1* F: TTGGCGACTACAAGACCACC; R: TGTACACAGGCACAGCAGAG, *Il6* F:AGAAAAGAGTTGTGCAATGG; R:ACAAACTCCAGGTAGAAACG, *Tnf* F:CCGTCCCTCTCATACACTGG; R: GGAACTTCTCCTCCTTGTTGG, *iNos* F: CAACATCAGGTCGGCCATTACT; R: TAGCCAGCGTACCGGATGA, *Mbp* F: ACACACAAGAACTACCCACTACGG; R: GTACGAGGTGTCACAATGTTCTTG.

### Statistical analysis

General statistical analyses were performed in GraphPad Prism software. Two group comparisons were throughout performed using Student’s two-tailed unpaired *t* test. Two group comparisons with a control group were analyzed using one-way ANOVA. Comparisons in EAE and stimulation with C75 were conducted with two-way ANOVA. *P* < 0.05 was throughout considered statistically significant. Analysis of RNA-seq data was performed using the DESeq2 package, which uses Wald testing with Benjamini–Hochberg adjustment for multiple testing.

### Reporting summary

Further information on research design is available in the Nature Research Reporting Summary linked to this article.

## Supplementary information

Supplementary Figures

Peer Review File

Reporting Summary

## Data Availability

Source data on RNA-seq used to generate graphs in Figs. 3 and 4 have been deposited in GEO database under accession number GSE154922. Additional data is available upon request via the corresponding author. Source data are provided with this paper.

## Source data

Source Data
