# Supplementary material for: Gsta4 controls apoptosis of differentiating adult oligodendrocytes during homeostasis and remyelination via the mitochondria-associated Fas-Casp8-Bid-axis
Source: Nat Commun. 2020 Aug 13;11:4071. doi: 10.1038/s41467-020-17871-5 (PMC7426940; doi:10.1038/s41467-020-17871-5)

Gsta4 controls apoptosis of differentiating adult oligodendrocytes during homeostasis and remyelination via the mitochondria-associated Fas-Casp8-Bid-axis

Carlström et al.

Supplementary Figure 1

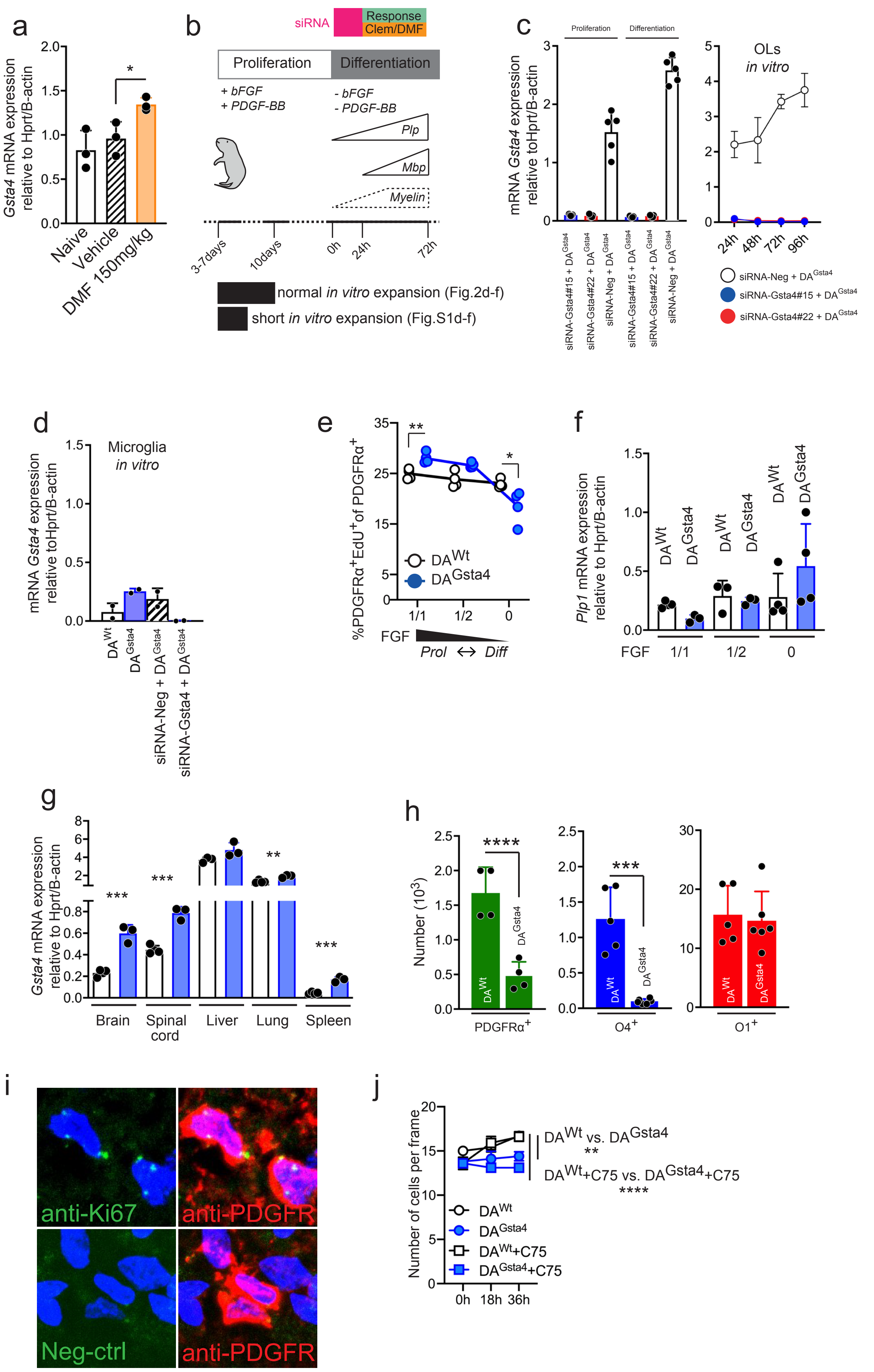

Supplementary Figure 2

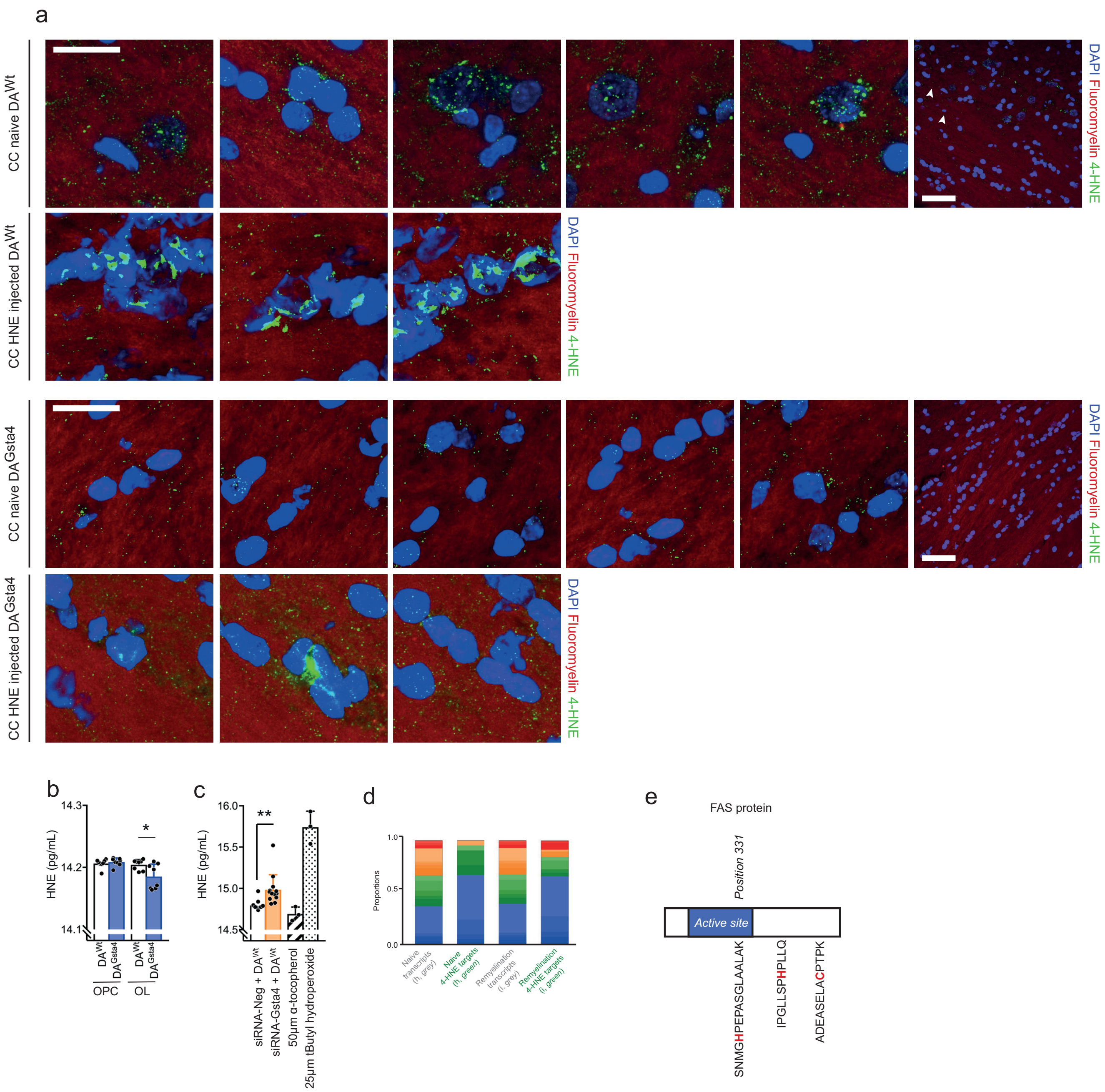

Supplementary Figure 3

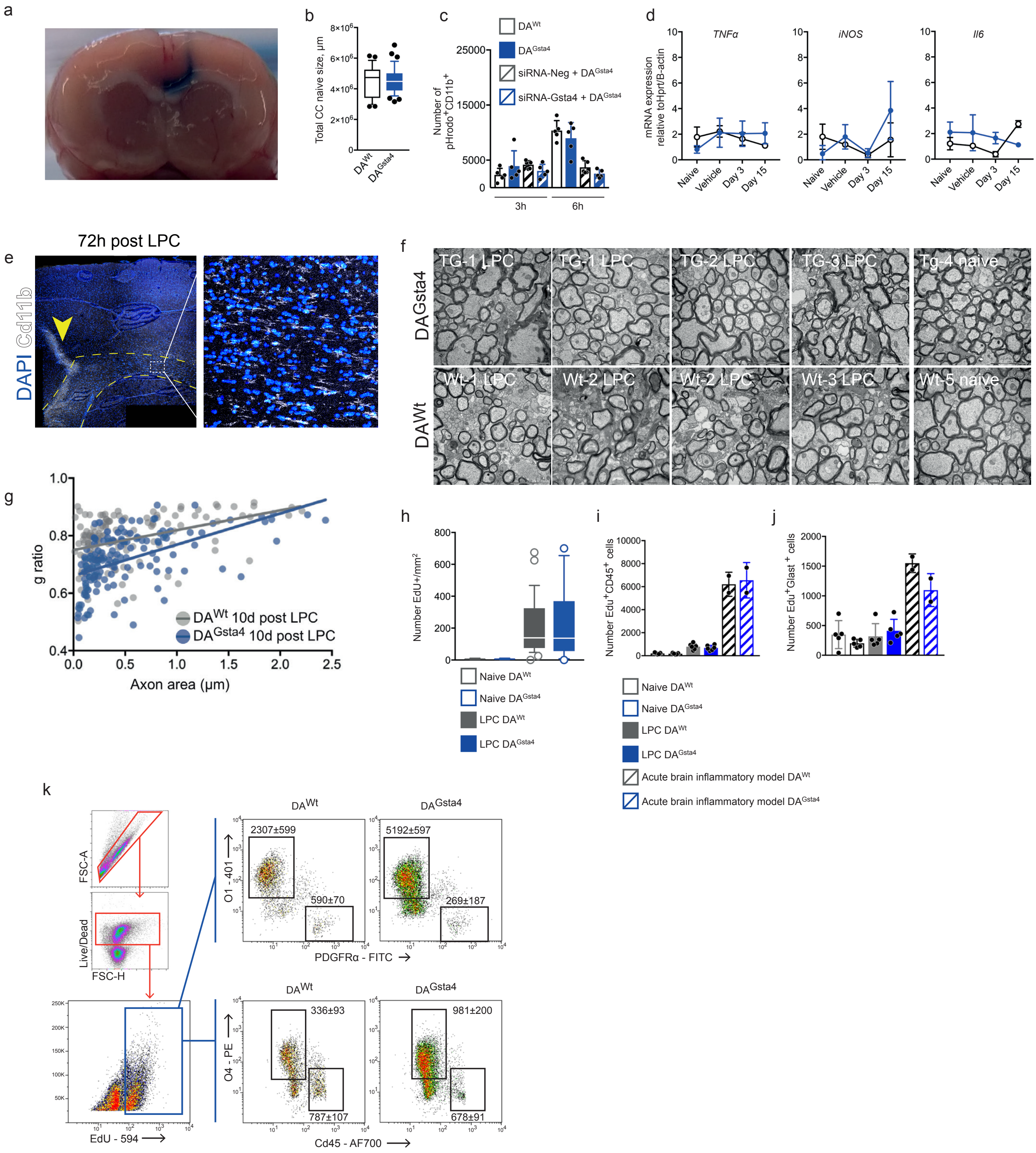

Supplementary Figure 4

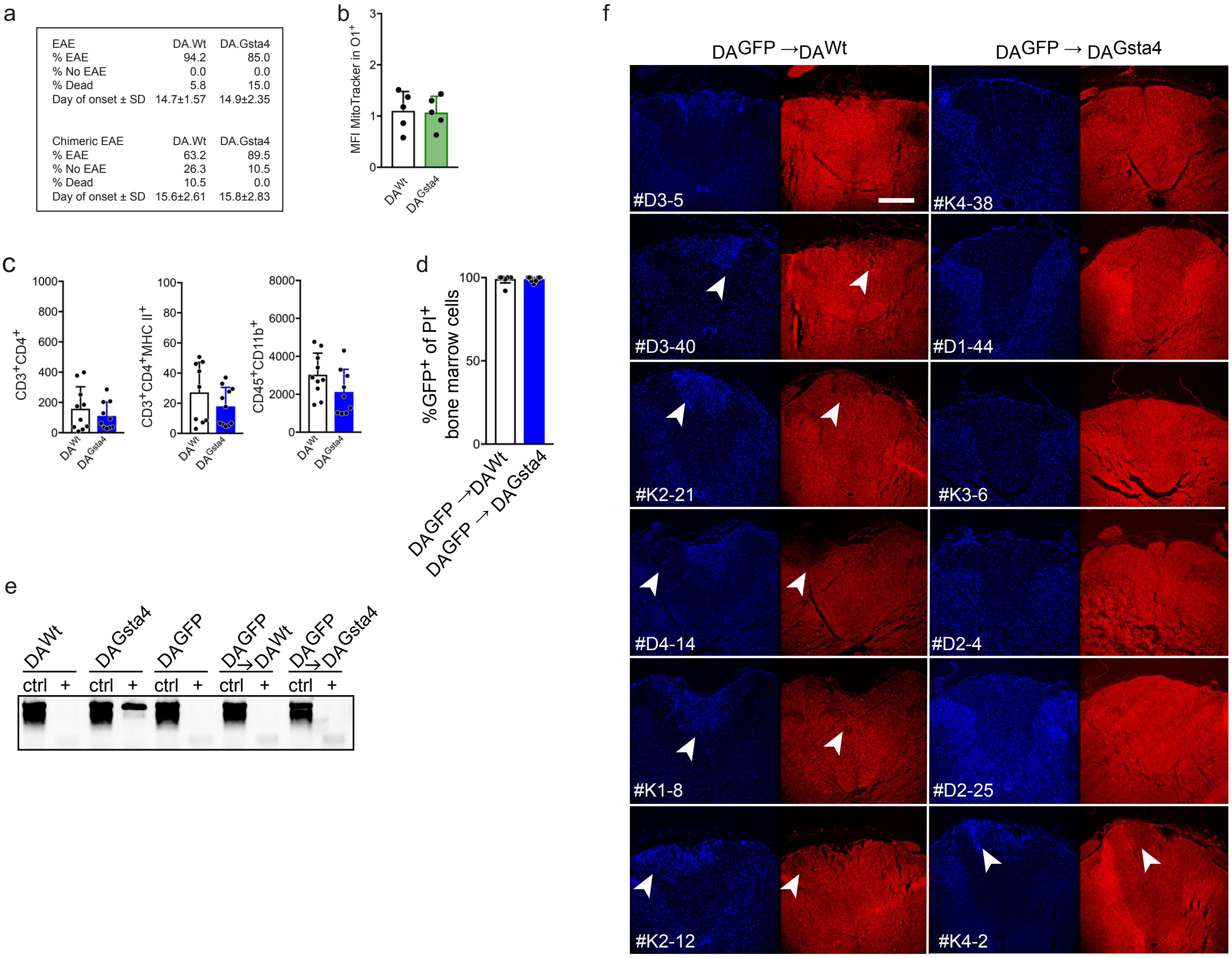

Supplement: Supplementary file 1 — Supplementary Figures [file 41467_2020_17871_MOESM1_ESM.pdf]
