# Supplementary material for: Gsta4 controls apoptosis of differentiating adult oligodendrocytes during homeostasis and remyelination via the mitochondria-associated Fas-Casp8-Bid-axis
Source: Nat Commun. 2020 Aug 13;11:4071. doi: 10.1038/s41467-020-17871-5 (PMC7426940; doi:10.1038/s41467-020-17871-5)
Supplement: Supplementary file 3 — Reporting Summary [file 41467_2020_17871_MOESM3_ESM.pdf]

nature research

Corresponding author(s): Karl Carlström

Last updated by author(s): Dec 19, 2019

Reporting Summary

Nature Research wishes to improve the reproducibility of the work that we publish. This form provides structure for consistency and transparency in reporting. For further information on Nature Research policies, see Authors & Referees and the Editorial Policy Checklist.

Statistics

For all statistical analyses, confirm that the following items are present in the figure legend, table legend, main text, or Methods section.

n/a	Confirmed	
☐	☒	The exact sample size (*n*) for each experimental group/condition, given as a discrete number and unit of measurement
☐	☒	A statement on whether measurements were taken from distinct samples or whether the same sample was measured repeatedly
☐	☒	The statistical test(s) used AND whether they are one- or two-sided<br>*Only common tests should be described solely by name; describe more complex techniques in the Methods section.*
☒	☐	A description of all covariates tested
☐	☒	A description of any assumptions or corrections, such as tests of normality and adjustment for multiple comparisons
☐	☒	A full description of the statistical parameters including central tendency (e.g. means) or other basic estimates (e.g. regression coefficient) AND variation (e.g. standard deviation) or associated estimates of uncertainty (e.g. confidence intervals)
☐	☒	For null hypothesis testing, the test statistic (e.g. *F*, *t*, *r*) with confidence intervals, effect sizes, degrees of freedom and *P* value noted<br>*Give P values as exact values whenever suitable.*
☒	☐	For Bayesian analysis, information on the choice of priors and Markov chain Monte Carlo settings
☒	☐	For hierarchical and complex designs, identification of the appropriate level for tests and full reporting of outcomes
☒	☐	Estimates of effect sizes (e.g. Cohen's *d*, Pearson's *r*), indicating how they were calculated

Our web collection on statistics for biologists contains articles on many of the points above.

Software and code

Policy information about availability of computer code

Data collection	*Provide a description of all commercial, open source and custom code used to collect the data in this study, specifying the version used OR state that no software was used.*
Data analysis	*Provide a description of all commercial, open source and custom code used to analyse the data in this study, specifying the version used OR state that no software was used.*

For manuscripts utilizing custom algorithms or software that are central to the research but not yet described in published literature, software must be made available to editors/reviewers. We strongly encourage code deposition in a community repository (e.g. GitHub). See the Nature Research guidelines for submitting code & software for further information.

Data

Policy information about availability of data

All manuscripts must include a data availability statement. This statement should provide the following information, where applicable:

- Accession codes, unique identifiers, or web links for publicly available datasets
- A list of figures that have associated raw data
- A description of any restrictions on data availability

The data that support the findings of this study are available from the corresponding author upon reasonable request.

Field-specific reporting

Please select the one below that is the best fit for your research. If you are not sure, read the appropriate sections before making your selection.

☒ Life sciences ☐ Behavioural & social sciences ☐ Ecological, evolutionary & environmental sciences

Life sciences study design

All studies must disclose on these points even when the disclosure is negative.

Sample size	No sample-size calculations were performed. Sample size was determined to be adequate based on the consistency of measurable differences between groups and were deemed sufficient to support the conclusion.
Data exclusions	No data were excluded from analysis.
Replication	All in vivo experiments were repeated at least 2 times, in vitro at least 3 times and results could be replicated each time.
Randomization	n/a
Blinding	Investigators were blinded during data collection from EAE, EdU and LPC experiments and analysis. Investigators were also blinded to associated IHC analysis.

Reporting for specific materials, systems and methods

We require information from authors about some types of materials, experimental systems and methods used in many studies. Here, indicate whether each material, system or method listed is relevant to your study. If you are not sure if a list item applies to your research, read the appropriate section before selecting a response.

Materials & experimental systems

n/a	Involved in the study	
☐	☒	Antibodies
☒	☐	Eukaryotic cell lines
☐	☐	Palaeontology
☐	☒	Animals and other organisms
☒	☐	Human research participants
☒	☐	Clinical data

Methods

n/a	Involved in the study	
☒	☐	ChIP-seq
☐	☒	Flow cytometry
☒	☐	MRI-based neuroimaging

Antibodies

Antibodies used

anti-PDGFRα (ab5460) (Abcam, Cambridge, GB) (1:100) (PubMedID:31171911)
anti-HNE (ab48506) (Abcam, Cambridge, GB) (1:50) (PubMedID:31040364)
anti-Plp (NB100-1608) (Novous Biologicals, Littleton, CO) (1:500) (PubMedID:26209807)
anti-CC1 (OP80) (Millipore, Darmstadt, Germany) (1:100) (PubMedID:8776583)
anti-Casp8 (NB100-56116) (Novous Biologicals, Littleton, CO) (1:200) (PubMedID:31151053)
Flow cytometry
anti-PDGFRα (KG215-7) (Creative Diagnostics; NY) (PubMedID:17215855)
anti-O4 (130-118-978) (Milteny Biotec, Bergisch, Germany) (PubMedID:2600978)
anti-O1 (FAB1327V) (Novous Biologicals, Littleton, CO) (PubMedID:9160244)
anti-CD45 (OX-1 (ROU)) (BD, Franklin Lakes, NJ) (PubMedID:7904617)

Validation

See PubMedID above.

Palaeontology

Specimen provenance	*Provide provenance information for specimens and describe permits that were obtained for the work (including the name of the issuing authority, the date of issue, and any identifying information).*
Specimen deposition	*Indicate where the specimens have been deposited to permit free access by other researchers.*
Dating methods	*If new dates are provided, describe how they were obtained (e.g. collection, storage, sample pretreatment and measurement), where they were obtained (i.e. lab name), the calibration program and the protocol for quality assurance OR state that no new dates are provided.*

☐ Tick this box to confirm that the raw and calibrated dates are available in the paper or in Supplementary Information.

Animals and other organisms

Policy information about studies involving animals; ARRIVE guidelines recommended for reporting animal research

Laboratory animals	Adult Dark Agouti rats. For neonatal OPC cultures both males and femals were used. Males were used throughout apart from EAE exp as female rats were used.
Wild animals	n/a
Field-collected samples	n/a
Ethics oversight	All experiments were approved and performed in accordance with Swedish National Board of Laboratory Animals and the European Community Council Directive (86/609/EEC) under the permits N275-15 and N244-13.

Note that full information on the approval of the study protocol must also be provided in the manuscript.

Flow Cytometry

Plots

Confirm that:

☒ The axis labels state the marker and fluorochrome used (e.g. CD4-FITC).

☒ The axis scales are clearly visible. Include numbers along axes only for bottom left plot of group (a 'group' is an analysis of identical markers).

☒ All plots are contour plots with outliers or pseudocolor plots.

☒ A numerical value for number of cells or percentage (with statistics) is provided.

Methodology

Sample preparation	Animals were sacrificed with carbon dioxide and perfused with PBS, the brain was removed and minced using a sterile scalpel. Tissues were processed as described for primary OPC cultures. Following enzymatic digestion, the homogenate was filtered through a 70m strainer and myelin was removed by a 37% Percoll layer spun at 800 x g for 10 min at +10°C without any brake.
Instrument	Samples were analyzed using with a 3-laser Beckman Coulter Gallios using Kaluza Software (Beckman Coulter, Brea, CA).
Software	Kaluza
Cell population abundance	The OPCs comprised 1-2% of all living cells based on Live/Dead stain.
Gating strategy	Single cells identified by FSH-A, FSH-H. Live/Dead stain (near IR Live/Dead (Thermo Fisher Scientific, Waltham, MA)). CD45 was used to exclude immune cells. Singel stains for every Ab and EdU was used to validate staining and to set up positive and negative stainings.

☒ Tick this box to confirm that a figure exemplifying the gating strategy is provided in the Supplementary Information.
