# Supplementary material for: Gsta4 controls apoptosis of differentiating adult oligodendrocytes during homeostasis and remyelination via the mitochondria-associated Fas-Casp8-Bid-axis
Source: Nat Commun. 2020 Aug 13;11:4071. doi: 10.1038/s41467-020-17871-5 (PMC7426940; doi:10.1038/s41467-020-17871-5)
Supplement: Supplementary file 2 — Peer Review File [file 41467_2020_17871_MOESM2_ESM.docx]

Peer Review Information

**Journal:** Nature Communications

**Manuscript title:** Gsta4 controls apoptosis of differentiating adult oligodendrocytes during homeostasis and remyelination via the mitochondria-associated Fas-Casp8-Bid-axis

**Corresponding author name(s):** Dr Karl Carlstrom

**Editorial notes:**

**none**

**Reviewer comments & decisions:**

**Reviewer comments, first version:**

Reviewer #1 (Remarks to the Author):

In the manuscript by Carlstrom et al., the authors investigated the effect of glutathione S transferase 4a (Gsta4) on oligodendrocytes, and found that Gsta4 regulates apoptosis in these cells via Fas/Casp8/Bid pathway. The investigators used a number of approaches to address the role of Gsta4 on oligodendrocytes in vitro and in vivo, as well as in demyelinating conditions including lysolecithin induced demyelination and EAE. However, there were typos and the experimental methods used seemed inconsistent/unfocused. At the same time, they were not rigorously performed. The use of DAgsta4 rats which displays global overexpression of Gsta4 in lysolecithin lesion vs. EAE raised more questions about what this gene does, and did not confirm its effect directly on oligodendrocyte lineage cells. Below are major points that should be addressed:

1. The figures were of low resolution, so it was challenging to see the images or text on the graphs clearly.

2. Although the effect of Gsta4 on oligodendrocytes in vitro was demonstrated, the conclusions made from in vivo experiments, that intrinsic Gsta4 function oligodendrocytes, was less convincing. Since the DA Gsta4 rat display overexpression throughout the entire animal, it remained possible that the DA Gsta4 overexpression affected metabolism/survival in many types of cells and affect OLs indirectly.

3. It was confusing exactly what Gsta4 does on oligodendrocyte lineage cells. The authors stated that Gsta4 overexpression (from OPC isolated from DAgsta4 rats) promoted enhanced proliferation of OPCs derived from neonatal pups compared to wild type conditions in vitro. In vivo, the authors found that in corpus callosum (CC), adult DAgsta4 was found to have a smaller pool of PDGFRα+ OPC (Fig.2b). However, the pool of OPCs was found to be more proliferative since a larger proportion of the DAGsta4 OPCs were positive for Ki67+ compared to DAWt (Fig.2c, d). The rats also displayed increased O4 and O1+ cells by flow cytometry, suggesting faster differentiation. But then found the rats did not exhibit increased mature CC1+ OLs by immunostaining (a graph was provided but it was odd that no images were shown). It was not clear if the immunostaing results (CC1) supported the flow cytometry data (O4 and O1). Moreover, if accelerated differentiation occurred as the authors suggested, why were mature CC1+ OL numbers not greater? Did the OPCs proliferate rapidly, accelerated differentiated, and then underwent apoptosis?

4. The analysis of Fas antagonist C75 on OL differentiation was performed by examining the number of OL branches in vitro, which differed from experiments performed in figure 1 which analyzed Mbp and Plp transcript expression. It was not clear why the authors did not use consistent analysis methods on the in vitro experiments.

5. Remyellination analysis was not rigorously performed in Fig 4. LFB staining of corpus callosum of lesioned DAgsta4 rat in Fig 4a was surprisingly intact. It was not clear if demyelination was actually achieved in this image. A better quality image should be provided to actually show demyelination.

6. Moreover, there was a lack of sufficient detail on the analysis and characterization of remyelination or OL lineage cell markers in lesions. Since the FACS analysis was performed on a large portion of the cortical tissue that includes both grey and white matter and corpus callosum (Fig 4i), lesion visualization by immunostaining and analysis with markers other than CC1 should be shown. And a smaller magnification image showing a lesioned corpus callosum would be important to confirm the labeling quantification in Fig. 4j.

7. The investigators stated that inflammation was unaffected or minimally affected in DAgsta4 rat by performing pHrodo-labeling in microglia culture after myelin addition (Fig.S1c, Fig.S3c), and by PCR analysis on bulk corpus callosum tissue. But in vivo immunostaining images of the lesion would have been informative, and was not done.

8. Figure S1 was absent or not displayed in the manuscript.

9. Figure 5. EAE does not tell you about remyelination and the data seemed to suggest that Gsta4 overexpression may be preventing demyelination (hence exhibit reduced demyelinated lesions) rather than stimulating remyelination. This point does not follow the premise of Gsta4 on oligodendrocyte lineage cell survival, and seemed to demonstrate the global effect of Gsta4 overexpression on metabolism rather than address its effect on oligodendrocytes directly. The relevance Gsta4 overexpression globally should be discussed.

10. The investigators suggested surprisingly that the levels of T-cells and macrophages were not significantly different from DAwt, but then transplantation of bone marrow (which is contain immune cells) from DAGgfp rat into DAgsta4 rat recapitulated EAE severity – which would suggest that immune cells are indeed involved. The investigators should elaborate this point, or perform more rigorous analysis of immune cell populations in EAE.

11. The investigators found that DAGsta4 rat with EAE also displayed smaller area of demyelination and fewer lesions in spinal cord dorsal column (Fig.5i, j). But staining of the spinal cord sections was poor, and lesions did not seem obvious in the images shown. Fig 5j seems to show images of the dorsal spinal cord, but EAE mainly affects the ventral cord. It was unclear why the dorsal cord was shown. Better immunostaining analysis should be provided to confirm there are indeed fewer lesions or demyelinated areas.

12. The premise of this study was based on the initial observation that dimethylfumarate (DMF) and clemastine influenced Nrf2 expression via Gsta4. However, no further experiments were performed to determine if DMF or clemastine regulated Gst4a expression or activity in the in vivo experiments (i.e. using lysolecithin, EAE, or DA Gsta4 rats). This manuscript would be stronger if the investigators show that DMF or clemastine treatment of DAwt resulted in similar phenotype or pathology as DAgsta4 rats.

13. Since Gsta4 has been described in astrocytes and other tissues, the global Gsta4 overexpression approach does not determine its role in directly regulating OL lineage cell progression.


Reviewer #3 (Remarks to the Author):

The manuscript by Carlström et al describes a novel pathway, glutathione S-transferase 4α (Gsta4) signaling, activated by dimethyl-fumarate (Tecfidera®) (DMF) and clemastine fumarate (CF), that is involved in oligodendrocyte precursor cell (OPC) maturation towards a myelinating phenotype and oligodendrocyte (OL) myelination both in vitro and in vivo. The authors provide elegant data on gain (GOF) and loss (LOF) functions, both genetically and pharmacologically, that provide strong evidence for their conclusions. Experiments are well designed and appropriately analyzed. Data conclusively show the involvement of the Gsta4 and Gsta4-4HNE pathways, as well as mitochondrial dysfunction leading to apoptosis, in OPC/OL differentiation. Importantly, clinicaltrials.gov lists 76 DMF and 1 CF MS trials ongoing. Thus, the potential clinical significance is high. Moreover, the depth with which the authors dissected the molecular signaling of this pathway is a major strength of this work. While DMF effects on OPCs/OLs have been previously reported by multiple laboratories (as noted by the authors), this is the first documentation of Gsta4 involvement in OPC/OL maturation.

There are concerns with this paper. The first is that the authors have not distinguished between the effects of the fumarte moiety and clemastine. There was no difference in efficacy between 30 um DMF and 15 um CF. The authors ” speculate that the fumarate molecule of CF is likely the cause of Nrf2 activation”, but this is not conclusively demonstrated. The signficance of this paper suffers in the absence of those data, as clemastine may have nothing to do with the observed effects. A second concern is the fact that these authors strongly interpret their data in the context of MS. However, mice models of MS and the course of the human disease differ in the fact that recent data about the latter involves argues for remyelination from adult OLs rather than differentiation from newly OPC-generated OL. There is controversy in the literature regarding this latter point, but it needs some discussion, especially when implicating clincial significance. Also lacking are data showing how Gsta4 knockdown affects DMF/CF-induced changes in OPC/OL function. There are likely other pathways affected by DMF/CF in OPC/OL and showing that the predominant biology is Gsta4-dependent would be important.

Minor issues are as follows:

I recognize the space limitations in the introduction, but at least one sentence detailing Gsta4 function is needed in that introduction to set the stage for the last paragraph of the intro as well as the rest of the paper. The authors can just move the phrase ”the main scavenger of 4-HNE” up one paragraph.

The methods do not indicate whether or not the heads were sheilded for X-irradiation. I assume not, but this should be stated, as it affects interpretation of brain inflammatory data.

There is no indication of the negative controls for immunohistochemistry. This is especially relevant for documentation of double immunoreactivity, which also should be shown with XZ and YZ planes provided.

What is the rationale for using 30 um DMF and 15 uM CF? As indicated above, current data do not provide any evidence that clemastine by itself is at all bioactive, as DMF and CF at these concentrations are indistinguishable in their actions. Ideally, they should be used at identical concentrations and with the addition of a non-fumarte derivative of clemastine to distinguish the actual biologically active moiety.

Why is DA WT different than DA WT + neg siRNA (Fig S1e)?

The authors show that DAWt branching was significantly increased upon addition of C75 (Fig.2k, l). What about C75 effects on cell number?

Pg 7, last paragraph: To make the statement that ”to have a reduced load of 4-HNE” requires quantitation of independent samples and proper statistics, not an empirical statement from a single sample.

Fig. 3f: Can’t have mean +/- SD with N=2; Fig 3h: Can’t do stats with N=2.

It appears that the DA Gsta4 group had a 3-fold higher mortality rate (5.8 vs 15.0) than the DA WT group. These data seem at odds with the statement that there was no difference in mortality between the groups. If this difference is real and significant, the authors should discuss this issue, or indicate otherwise.

Throughout the manuscript, there are minor grammatical errors with respect to appropriate verb usage and lack of necessary articles. I suggest a native English speaker be asked to clean up the grammar.

**Author rebuttal, first version:**

Reviewer #1 (Remarks to the Author): In the manuscript by Carlstrom et al., the authors investigated the effect of glutathione S transferase 4a (Gsta4) on oligodendrocytes, and found that Gsta4 regulates apoptosis in these cells via Fas/Casp8/Bid pathway. The investigators used a number of approaches to address the role of Gsta4 on oligodendrocytes in vitro and in vivo, as well as in demyelinating conditions including lysolecithin induced demyelination and EAE. However, there were typos and the experimental methods used seemed inconsistent/unfocused. At the same time, they were not rigorously performed. The use of DAgsta4 rats which displays global overexpression of Gsta4 in lysolecithin lesion vs. EAE raised more questions about what this gene does, and did not confirm its effect directly on oligodendrocyte lineage cells. Below are major points that should be addressed:

Reviewer #1 The figures were of low resolution, so it was challenging to see the images or text on the graphs clearly.

**Reply:** We apologize for the poor resolution in the initial version. When we observed this we contacted the editorial office and provided a new higher resolution version. High resolution images are provided together with the revised manuscript file.

Reviewer #1 The premise of this study was based on the initial observation that dimethylfumarate (DMF) and clemastine influenced Nrf2 expression via Gsta4. However, no further experiments were performed to determine if DMF or clemastine regulated Gst4a expression or activity in the in vivo experiments (i.e. using lysolecithin, EAE, or DA Gsta4 rats). This manuscript would be stronger if the investigators show that DMF or clemastine treatment of DAwt resulted in similar phenotype or pathology as DAgsta4 rats.

**Reply:** The DMF-mediated effect on Nrf2 is well described (PubMedID: 21354971), also in oligodendrocytes (PubMedID: 25967672), and we have previously described a downstream effect on Gsta4.

The role of Clemastine in OL cultures (PubmedID: 24997607, 30046099), demyelinating experimental models (PubmedID: 26253956, 27671734, 29244098, 29584714) and in human clinical trials (PubmedID: 29029896, 29029894) has already been well established. However, as described above it is not always clear exactly what clemastine formulation was used in some of the mentioned studies. Based on the in vitro data presented in the revised manuscript we show that Clemastine fumarate (Clem-F) has an effect profile resembling that of DMF, while clemastine hydrochloride (Clem-H) displayed a weaker effect. Importantly, the clinical trial in MS patients was conducted with Clem-F and we believe that further dissection of possible differences between the two formulations of clemastine is out of the scope of this study. We have now added data on Gsta4 transcription in brain tissue following oral gavage with DMF in DAwt rats.

***Change in text:***

*“Following oral gavage DMF also elevated Gsta4 transcription in brain tissue following oral gavage (Fig.S1a).”*

Reviewer #1 Figure S1 was absent or not displayed in the manuscript.

**Reply:** We apologize for the poor resolution in the uploaded version. When we observed this we contacted the editorial office and provided a high resolution version. High resolution images was originally also provided in addition to the manuscript file.

Reviewer #1 It was confusing exactly what Gsta4 does on oligodendrocyte lineage cells. The authors stated that Gsta4 overexpression (from OPC isolated from DAgsta4 rats) promoted enhanced proliferation of OPCs derived from neonatal pups compared to wild type conditions in vitro. In vivo, the authors found that in corpus callosum (CC), adult DAgsta4 was found to have a smaller pool of PDGFRα+ OPC (Fig.2b). However, the pool of OPCs was found to be more proliferative since a larger proportion of the DAGsta4 OPCs were positive for Ki67+ compared to DAWt (Fig.2c, d).

**Reply:** We agree with the reviewer that the effects of Gsta4 on proliferation appear to be context dependent. We found that DA^Gsta4^ OPCs expanded shortly after neonatal isolation proliferated more ex vivo compared to DA^Wt^ OPCs. However, when expanding the OPCs in vitro for longer time periods (10days) in the presence of bFGF we instead observed more proliferation in DA^Wt^ OPCs . This was not the case upon differentiation (absence of bFGF), where there was a similar decrease of proliferative DA^Gsta4^ OPCs compared to DA^Wt^. This suggests that longer exposure of OPCs to bFGF might prime them to differentiate. In vivo, despite a smaller OPC pool in the DA^Gsta4^, a similar number of double-positive PDGFRα^+^Ki67^+^ was observed in the corpus callosum (Fig.3d, Fig.S1h), which could suggest a compensatory mechanism to compensate for the smaller proportion of OPCs, possibly involving bFGF (Fig.S1e). These data are now presented in Fig.2d-f and Fig.S1e-f). The experimental set-up is also illustrated in Fig.S1b.

***Change in text:*** *Results Page 6*

*“In concordance with previous in vitro findings (Fig.2) adult DA^Gsta4^ had fewer OLs identified using IHC staining of PDGFRα^+^ (Fig.3b). Flow cytometric analysis of corpus callosum and grey matter regions (indicated in Fig.3a, dotted line) corroborated these histological findings evident by decreased numbers of PDGFRα^+^ OPCs and pre-mature O4^+^ OLs in DA^Gsta4^ compared to DA^Wt^ (Fig.S1h). Despite a smaller OPC pool in the DA^Gsta4^ stain a similar number of double-positive PDGFRα^+^Ki67^+^ was observed in the corpus callosum (Fig.3d, Fig.S1i), which could suggesting a compensatory mechanism for OPC loss and possibly involving bFGF (Fig.S1e).”*

***Change in text:*** *Results Page 5*

*“Basic fibroblast growth factor (bFGF) is essential in OPC in vitro expansion, as it promotes proliferation and blocks differentiation (Fig.S1b). Upon withdrawal of bFGF, the DA^Wt^ and DA^Gsta4^ OPC pools differentiated. However, DA^Gsta4^ expressed higher levels of Plp1 both in the presence of bFGF during extended in vitro expansion, and also in its absence upon differentiation (Fig.2d). Accordingly, there was a lower degree of proliferative (PDGFRα^+^EdU^+^/PDGFRα^+^) DA^Gsta4^ OPCs compared to DA^Wt^ OPCs in both presence or absence of bFGF (Fig.2e). Nevertheless, upon short in vitro expansion (Fig. S1b), while there was a similar decrease of proliferative DA^Gsta4^ OPCs upon differentiation (no bFGF) compared to DA^Wt^, there was a slightly higher percentage of PDGFRα^+^EdU^+^/PDGFRα^+^ in DA^Gsta4^ compared to DA^Wt^ and decreased Plp1 when bFGF was present (Fig.S1e, f). This suggests that long-term exposure of OPCs to bFGF might prime them to respond by triggering differentiation upon Gsta4 over-expression.”*

Reviewer #1 Although the effect of Gsta4 on oligodendrocytes in vitro was demonstrated, the conclusions made from in vivo experiments, that intrinsic Gsta4 function oligodendrocytes, was less convincing. Since the DA Gsta4 rat display overexpression throughout the entire animal, it remained possible that the DA Gsta4 overexpression affected metabolism/survival in many types of cells and affect OLs indirectly.

Since Gsta4 has been described in astrocytes and other tissues, the global Gsta4 overexpression approach does not determine its role in directly regulating OL lineage cell progression.

**Reply:** This is a valid comment that we have tried to address using various approaches.

The OL-specificity of an Gsta4 effect is also supported by a lack of difference in astrocyte phenotype between the two genotypes, as shown by data on astrocyte numbers in the LPC model. Further, the absence of a microglia phenotype in Gsta4 animals also does not support the notion of a more generalized effect on the glial/microglial lineage.

***Change in text:*** *Results Page 11*

*“Neither, no strain differences in numbers of immune cells or astrocytes following LPC administration were observed (Fig.S3e, h-j). Collectively, this indicates that Gsta4 over-expression had no or only a negligible effect on the inflammatory response in the LPC model.”*

***Change in text:*** *Figure legend* *Fig.S3*

*“(i) Number of double-positive CD45+EdU+ cells during homeostasis, ten days following LPC injections or application of traumatic brain injury model. (j) Number of double-positive Glast+EdU+ cells during homeostasis, ten days following LPC injections or application of traumatic brain injury model.”*

Reviewer #1 The rats also displayed increased O4 and O1+ cells by flow cytometry, suggesting faster differentiation. But then found the rats did not exhibit increased mature CC1+ OLs by immunostaining (a graph was provided but it was odd that no images were shown). It was not clear if the immunostaing results (CC1) supported the flow cytometry data (O4 and O1).

**Reply:** We agree with the reviewer. Consistent with these findings, we did not record differences in O1+ OLs either. Thus, observations with immunostaining and flow data are in agreement (Fig.3g, FigS1h red). However, while we observed faster differentiation PDGFRa+ and O4+ OLs were decreased in DAGsta4 compared to DAWT, as shown by immunohistochemistry and flow cytometry (Fig.3b-d, Fig.S1i). It is possible that a compensation between proliferation and differentiation rates might contribute to the homeostatic levels of OLs in DA^Gsta4^. In addition, our data also suggests that other cellular mechanisms as cell survival might also be involved. We have now discussed these possibilities in the manuscript.

***Change in text:*** *Results page 6*

*“Despite a smaller OPC pool in the DA^Gsta4^ stain a similar number of double-positive PDGFRα^+^Ki67^+^ was observed in the corpus callosum (Fig.3d, Fig.S1i), which could suggesting a compensatory mechanism for OPC loss and possibly involving bFGF (Fig.S1e)* .”

***Change in text:*** *Results page 7*

*“While a compensation between proliferation and differentiation rates might contribute to the homeostatic levels of OLs in DAGsta4, this finding suggested that other cellular mechanisms might also be involved.”*

Reviewer #1 Moreover, if accelerated differentiation occurred as the authors suggested, why
were mature CC1+ OL numbers not greater? Did the OPCs proliferate rapidly, accelerated differentiated, and then underwent apoptosis?

**Reply:** We agree with the reviewer that it is possible that a inter-dependent regulation of proliferation and differentiation rates might contribute to the homeostatic levels of OLs in DA^Gsta4^. Our data indicate that differentiation of DAGsta4 OPCs is accelerated compared to DAWt. Together with the fact that fewer OPCs reached a mature state after 6 weeks, a time point sufficiently long for OPC to differentiate to mature OL, in DAWT compared to DAGsta4 (Fig.3e, f), this suggests more OPCs being lost during differentiation in DAWt compared to DAGsta4.

***Change in text:*** *Results Page 7*

*“The proportions of both double-positive O4+EdU+ and O1+EdU+ of all O4+ or O1+ were higher in DAGsta4 at both ten days and six weeks (Fig.3e, f) suggesting a faster pace of differentiation in DAGsta4. However, the total number of CC1+ OLs in the adult corpus callosum was not different between DAGsta4 and DAWt (Fig.3g, Fig.S1h). While a compensation between proliferation and differentiation rates might contribute to the homeostatic levels of OLs in DAGsta4, this finding suggested that other cellular mechanisms might also be involved.*

*To identify pathways contributing to the observed phenotype O4+ OLs were sorted out from micro-dissected adult DAWt (n=5) and DAGsta4 (n=5) corpus callosum followed by RNA-seq (Fig.3a).”*

Reviewer #1 The analysis of Fas antagonist C75 on OL differentiation was performed by examining the number of OL branches in vitro, which differed from experiments performed in figure 1 which analyzed Mbp and Plp transcript expression. It was not clear why the authors did not use consistent analysis methods on the in vitro experiments.

**Reply:** Verifying qPCR data has been added (Fig.3m).

***Change in text:*** *Results Page 7*

*“DA^Wt^ branching and Plp1 expression were significantly increased upon addition of C75 (Fig.3k-m, Fig.S1j).”*

Reviewer #1 Remyellination analysis was not rigorously performed in Fig 4. LFB staining of corpus callosum of lesioned DAgsta4 rat in Fig 4a was surprisingly intact. It was not clear if demyelination was actually achieved in this image. A better quality image should be provided to actually show demyelination.

**Reply:** Luxol fast blue staining in Fig5a shows myelin at day 15. Based on prior experience and existing literature it is not surprising that while the corpus callosum is remyelinated in DAGsta4, DAWt shows a clear lesion core. To address the reviewer’s concern we have thus also added the representative images on which the time-laps graph is based on.

***Change in text:*** *Results Page 10*
*“While Luxol fast blue (LFB) staining initially revealed a similar degree of demyelination between the two strains, examinations at later time-points revealed a more efficient remyelination in DA^Gsta4^ compared to in DA^Wt^ (Fig.5b). The difference between strains was already evident at 72h and persisted until 15 days post injection, a time point when DA^Gsta4^ animals displayed minimal visible demyelinating damage, whereas such damage was still readily evident in the DA^Wt^ strain.”*

***Change in text:*** *Results Page 9*

*“A two-fold increase in the percentage of myelinated axons was recorded in DA^Gsta4^ compared to DA^Wt^ during remyelination (Fig.5c, d), where a difference in myelin thickness was primarily evident in thinner axons (Fig.S3f, g). The G-ratio describes the diameter of the transected axon in relation to the outer diameter of the surrounding myelin as assessed using TEM. Using a software that randomly selects axons for analysis from every image frame^59^, DA^Wt^ axons were determined to be less myelinated after remyelination compared to DA^Gsta4^ axons (Fig.5c, e, Fig.S3f). A small but significant difference was also evident between the strains during homeostasis, as DA^Gsta4^ axons had thinner myelin. We have previously reported that myelin sheath thickness correlates with axonal mitochondria size and that this is an indicator of advanced remyelination^60^, and we herein observed that at day ten DA^Gsta4^ animals re-established this correlation during homeostasis while DA^Wt^ did not (Fig.5f).”*

Reviewer #1 Moreover, there was a lack of sufficient detail on the analysis and characterization of remyelination or OL lineage cell markers in lesions. Since the FACS analysis was performed on a large portion of the cortical tissue that includes both grey and white matter and corpus callosum (Fig 4i), lesion visualization by immunostaining and analysis with markers other than CC1 should be shown. And a smaller magnification image showing a lesioned corpus callosum would be important to confirm the labeling quantification in Fig. 4j.

**Reply:** This is a relevant point. Unfortunately, commercially available Abs for O1 and O4 perform poorly on rat brain sections. However, our TEM analysis of the corpus callosum are consistent with our flow cytometric analyses.

Reviewer #1 The investigators stated that inflammation was unaffected or minimally affected in DAgsta4 rat by performing pHrodo-labeling in microglia culture after myelin addition (Fig.S1c, Fig.S3c), and by PCR analysis on bulk corpus callosum tissue. But in vivo immunostaining images of the lesion would have been informative, and was not done.

***Reply:*** pHrodo-experiments was performed to exclude an effect of Gsta4 on microglial phagocytic capacity, important for myelin debris scavenging and for ensuing remyelination. We have in addition added Cd11b+ IHC images of corpus callosum to better visualize microglia following LPC (Fig.S3e). In addition, quantification of microglia (Cd45+) and astrocyte (Glast+) was performed with flow cytometry and provided in FigS3i, j.

**Change in text:** Results page 10

*“Since incomplete scavenging of myelin debris is known to limit OPC differentiation and remyelination, the possible contribution from Gsta4 in microglia during phagocytosis was assessed. pHrodo-labeled myelin was added to primary microglia cultures but no difference in phagocytosis at any time-point between DA^Wt^ and DA^Gsta4^ or siRNA-Neg and siRNA-Gsta4 was observed (Fig.S1c, Fig.S3c). Moreover, transcription of pro-inflammatory hallmark genes did not vary in bulk corpus callosum tissue at any time point following LPC exposure (Fig.S3d). Neither, was any strain differences in numbers of immune cells or astrocytes following LPC administration observed (Fig.S3e, h-j). Collectively, this indicates that Gsta4 over-expression had no or only a negligible effect on the inflammatory response in the LPC model.”*


Reviewer #1 Figure 5. EAE does not tell you about remyelination and the data seemed to suggest that Gsta4 overexpression may be preventing demyelination (hence exhibit reduced demyelinated lesions) rather than stimulating remyelination. This point does not follow the premise of Gsta4 on oligodendrocyte lineage cell survival, and seemed to demonstrate the global effect of Gsta4 overexpression on metabolism rather than address its effect on oligodendrocytes directly. The relevance Gsta4 overexpression globally should be discussed.

**Reply:** We agree with the reviewer and have discussed in the text that the effects observed might be a result of decreased demyelination or increased remyelination (given the results from the LPC model). To further address the reviewer’s concerns and link our previous findings on Fas/Casp8/Bid-axis during oligodendrocyte differentiation we repeated EAE experiments and analyzed mRNA from spinal cord O4+ OLs. We again found Bid to be lowered in DAGsta4 OLs.

***Change in text:*** *Results page 12*

*“There were no differences in terms of disease incidence or day-of-onset between the groups (Fig.S4a), suggesting that the triggering of an autoimmune response did not differ between the strains. However, after the onset of disease animals over-expressing Gsta4 displayed a milder disease course (Fig.6a), with reduced disease duration and cumulative disability scores compared to DA^Wt^ (Fig.6b). As observed in corpus callosum O4^+^ OLs during homeostasis (Fig.3i), mRNAs of mediators partaking in the Fas/Casp8/Bid-axis were also differentially regulated in the spinal cord O4^+^ OLs comparing DA^Gsta4^ and DA^Wt^ at day 42 after immunization (Fig.6c). In agreement with this, at the end of experimental follow up, the MitoSOX signal in O1^+^ was lower in DA^Gsta4^ compared to in DA^Wt^ (Fig.6e, Fig.S4b).”*

Reviewer #1 The investigators suggested surprisingly that the levels of T-cells and macrophages were not significantly different from DAwt, but then transplantation of bone marrow (which is contain immune cells) from DAGgfp rat into DAgsta4 rat recapitulated EAE severity – which would suggest that immune cells are indeed involved. The investigators should elaborate this point, or perform more rigorous analysis of immune cell populations in EAE.

**Reply:** We are sorry for not providing sufficient detail about this particular experiment. In order to inform the readers about the basic principles of bone marrow transfer in EAE, we have added an illustration in the figure and made some clarifications in the text. In brief, the transplanted bone marrow cells do not have increased expression of Gsta4, so any observed effect can be attributed to the recipient resident CNS cells, expressing higher levels of Gsta4.

***Change in text:*** *Results page 12*

*“We could not observe any differences in the numbers of infiltrating leukocytes or activated microglia in the spinal cords between DA^Gsta4^ and DA^Wt^ strains (Fig.S4c), suggesting that Gsta4 did not either affect adaptive or innate immunity.*

*In order to further substantiate this notion we repeated EAE experiments in bone marrow-transplanted DA^Wt^ and DA^Gsta4^ (Fig.S4d, e). Animals were lethally irradiated without any shielding and transplanted with green fluorescent protein (GFP)-expressing bone marrow cells from DA^GFP^ followed by immunization with MOG in adjuvant (Fig.6e). DA^Gsta4^ animals transplanted with DA^GFP^ bone marrow (DA^GFP^→DA^Gsta4^) recapitulated the EAE phenotype previously observed in DA^Gsta4^ (Fig.6f). Disease duration and maximal disease scores were significantly lower in DA^GFP^→DA^Gsta4^ compared to in DA^GFP^→DA^Wt^ (Fig.6g). In concordance with decreased mRNA transcription of Fas/Casp8/Bid-axis mediators and decreased mitochondrial stress in DA^Gsta4^ compared to in DA^Wt^, DA^GFP^→DA^Gsta4^ exhibited lower levels of active Casp8 staining in CC1^+^ spinal cord (Fig.6h, i). This resulted in a smaller area of demyelination and fewer lesions in the spinal cord dorsal column in DA^Gsta4^ compared to in DA^Wt^ (Fig.6j-l, FigS4f).”*

Reviewer #1 The investigators found that DAGsta4 rat with EAE also displayed smaller area of demyelination and fewer lesions in spinal cord dorsal column (Fig.5i, j). But staining of the spinal cord sections was poor, and lesions did not seem obvious in the images shown. Fig 5j seems to show images of the dorsal spinal cord, but EAE mainly affects the ventral cord. It was unclear why the dorsal cord was shown. Better immunostaining analysis should be provided to confirm there are indeed fewer lesions or demyelinated areas.

**Reply:** The reason for choosing the dorsal column was based on two aspects: (1) inflammatory immune cell infiltrates in MOG-EAE are common in the dorsal column (PubmedID: 26207625), and (2) it has a distinct appearance with clear delineation, which facilitates quantification along the spinal cord in EAE. Related images have been changed.

***Change in text*** *Results page 13*

*“In concordance with decreased mRNA transcription of Fas/Casp8/Bid-axis mediators and decreased mitochondrial stress in DA^Gsta4^ compared to in DA^Wt^, DA^GFP^→DA^Gsta4^ exhibited lower levels of active Casp8 staining in CC1^+^ spinal cord (Fig.6h, i). This resulted in a smaller area of demyelination and fewer lesions in the spinal cord dorsal column in DA^Gsta4^ compared to in DA^Wt^ (Fig.6j-l, FigS4f).”*

Reviewer #3 (Remarks to the Author): The manuscript by Carlström et al describes a novel pathway, glutathione S-transferase 4α (Gsta4) signaling, activated by dimethyl-fumarate (Tecfidera®) (DMF) and clemastine fumarate (CF), that is involved in oligodendrocyte precursor cell (OPC) maturation towards a myelinating phenotype and oligodendrocyte (OL) myelination both in vitro and in vivo. The authors provide elegant data on gain (GOF) and loss (LOF) functions, both genetically and pharmacologically, that provide strong evidence for their conclusions. Experiments are well designed and appropriately analyzed. Data conclusively show the involvement of the Gsta4 and Gsta4-4HNE pathways, as well as mitochondrial dysfunction leading to apoptosis, in OPC/OL differentiation. Importantly, clinicaltrials.gov lists 76 DMF and 1 CF MS trials ongoing. Thus, the potential clinical significance is high. Moreover, the depth with which the authors dissected the molecular signaling of this pathway is a major strength of this work. While DMF effects on OPCs/OLs have been previously reported by multiple laboratories (as noted by the authors), this is the first documentation of Gsta4 involvement in OPC/OL maturation.

Reviewer #3 Throughout the manuscript, there are minor grammatical errors with respect to appropriate verb usage and lack of necessary articles. I suggest a native English speaker be asked to clean up the grammar.

***Reply:*** We agree. The experiment have been repeated to allow for proper statistics.

Reviewer #3 There are concerns with this paper. The first is that the authors have not distinguished between the effects of the fumarte moiety and clemastine. There was no difference in efficacy between 30 um DMF and 15 um CF. The authors ” speculate that the fumarate molecule of CF is likely the cause of Nrf2 activation”, but this is not conclusively demonstrated. The signficance of this paper suffers in the absence of those data, as clemastine may have nothing to do with the observed effects.

**Reply:** We agree with the reviewer that this is an important point. In order to address this we have compared Clemastine-fumarate (Clem-F) to Clemastine-HCl (Clem-H) using the same concentrations. Hence, we have identified Clem-F as having a broader therapeutic spectrum of effects in vitro compared to Clem-H and clearly the fumarate moiety in Clem-F contributes to enhanced maturation via transcription of Plp (Fig.1b, c). In addition Clem-F, but not Clem-H, was able to activate Nrf2 to a similar extent as DMF compared to DMSO controls (Fig.1d). Collectively, we cannot exclude that clemastine exerts some effects by itself, but our observations clearly suggest that the fumarate moeity provides additional effects.

***Change in text:*** *Results Page 4*

*“In order to elucidate the contribution of the fumarate moiety we evaluated the effect of clemastine fumarate (Clem-F) and clemastine hydrochloride (Clem-H) (Fig.1b). Both of these increased Plp1 transcription, but the effect of the latter was significantly greater (Fig.1c).*

*The transcription factor Nrf2 has been identified as the main therapeutic target of dimethyl fumarate (DMF) in several cell types^40,46-48^. Electrophilic agents such as DMF and its active metabolites (e.g monomethyl fumarate (MMF)) facilitates Nrf2 activation via Keap1^49,50^. We also determined that DMF and Clem-F caused nuclear activation of Nrf2 in OLs to a larger extent than both control and Clem-HCl treated OLs (Fig.1d). Interestingly, Gsta4, the main scavenger of the lipid peroxidation product 4-hydroxynonenal (4-HNE), has been predicted to be a target of Nrf2^51,52^, which we corroborated through the observation that both DMF and Clem-F induced the expression of Gsta4 (Fig.1e). Following oral gavage DMF also elevated Gsta4 transcription in brain tissue following oral gavage (Fig.S1a). Furthermore, Gsta4 transcription was strongly increased in OLs during differentiation compared to in proliferating OPCs (Fig.1f).”*

Reviewer #3 What is the rationale for using 30 um DMF and 15 uM CF? As indicated above, current data do not provide any evidence that clemastine by itself is at all bioactive, as DMF and CF at these concentrations are indistinguishable in their actions. Ideally, they should be used at identical concentrations and with the addition of a non-fumarte derivative of clemastine to distinguish the actual biologically active moiety.

**Reply:** The clemastine moiety of the clemastine formulation has previously been ascribed to promote myelinating features via muscarinic receptors (PubMedID: 27671734). There is also an unfortunate semantic simplification in the literature where it is not always obvious if “clemastine” refers to “clemastine fumarate” or formulations without fumarate. We do not think that our observations disagree with a beneficial role of clemastine in remyelination, however, in our set up fumarate clearly provided additional effects not seen with a clemastine formulation without this moiety.

The tolerability of DMF by primary cells and cell lines is relatively high, as we have shown before (PubMedID:26415122, 32394330). Clem-F has been tested at the concentration previously described (PubmedID: 24997607, 30046099) and the same concentration was used for Clem-HCl. The initial experiments have now been repeated using the same concentration for DMF, Clem-F and Clem-H (10uM).

Reviewer #3 Also lacking are data showing how Gsta4 knockdown affects DMF/CF-induced changes in OPC/OL function. There are likely other pathways affected by DMF/CF in OPC/OL and showing that the predominant biology is Gsta4-dependent would be important.

**Reply:** The lack of Gsta4 have been shown to trigger increased oxidative stress, toxicity and apoptosis (PubMedID: 14761685, 24353929, 21425933). Even if Nrf2 is considered the main target for DMF, additional targets have been suggested, as shown by us and others (PubmedID: 31300673, 26415122, 29599194). Nrf2-activation is preceded by oxidative stress, where Gsta4 has been shown to be protective. One could thus speculate that cells lacking Gsta4 generally would be more vulnerable to any treatment or stimulation that leads to increased oxidative stress. We herein show that that lack of Gsta4 (by using siRNA) affects differentiation (Fig.2g-i) and that DMF and Clem-F work in a Gsta4-dependent manner to promote differentiation (Fig.1g). Lastly, our data on mitochondrial stress due to lack on Gsta4 overlaps well with the current understanding (Fig.4g).

Reviewer #3 Why is DA WT different than DA WT + neg siRNA (Fig S1e)?

**Reply:** Results relating to use of siRNA are not reported in Fig.S1e. However, upon the use of siRNA, cells are transfected using lipofectamine, a procedure which is known to affect cells by itself. To control for such effects control samples only treated with lipofectamine without siRNA have been used. (e.g. Fig.1g). Fig.S1b illustrates siRNA efficiency over time and in comparison to an alternative siRNA towards Gsta4.

Reviewer #3 The authors show that DAWt branching was significantly increased upon addition of C75 (Fig.2k, l). What about C75 effects on cell number?

***Reply*:** C75 had no effect on cell number in neither DAWt or Gsta4 OPCs. This has been added. (Fig.S1j)

***Change in text:*** *Results Page 7*

*“DA^Wt^ branching and Plp1 expression were significantly increased upon addition of C75 (Fig.3k-m, Fig.S1j).”*

Reviewer #3 Pg 7, last paragraph: To make the statement that ”to have a reduced load of 4-HNE” requires quantitation of independent samples and proper statistics, not an empirical statement from a single sample.

***Reply*:** We have now provided the requested information on how many animals were used. This has been addressed in FigS2a and Fig.S2 legend

**Change in text:** Fig.S2 legend

*a) Representative confocal images of anti-4-HNE staining of naïve CC from DAWt and DAGsta4 (n=3) and following intraparenchymal injections of HNE (n=1).*

Reviewer #3 Fig. 3f: Can’t have mean +/- SD with N=2; Fig 3h: Can’t do stats with N=2.

***Reply*:** We agree. The experiment have been repeated with more ns in order to perform proper statistics.

Reviewer #3 There is no indication of the negative controls for immunohistochemistry. This is especially relevant for documentation of double immunoreactivity, which also should be shown with XZ and YZ planes provided.

***Reply:*** This has been addressed in Fig3, Fig5j,Fig.S1 and Fig.S4.

Reviewer #3 A second concern is the fact that these authors strongly interpret their data in the context of MS. However, mice models of MS and the course of the human disease differ in the fact that recent data about the latter involves argues for remyelination from adult OLs rather than differentiation from newly OPC-generated OL. There is controversy in the literature regarding this latter point, but it needs some discussion, especially when implicating clincial significance.

**Reply:** We agree with the reviewer that more research is needed to better understand to what degree results can be extrapolated to human conditions. However, we believe a strength with this paper is the use of an EAE model in rat that show more similarities with MS than most mice models (PubMed ID:9804377). However, there are papers clearly describing differences between species and this has been added to the discussion.

**Change in text:** *Discussion page 15*

*“Finally, we observed that Gsta4 over-expression leads to behavioral amelioration upon induction of MS-like disease (EAE). Several reports have suggested differences between species regarding adult remyelination^18,65,66^, and EAE is a widely accepted model for MS-like disease in rodents.”*

Reviewer #3The methods do not indicate whether or not the heads were sheilded for X-irradiation. I assume not, but this should be stated, as it affects interpretation of brain inflammatory data.

**Reply:** This has been addressed

***Change in text:*** *Results Page 12*

“Animals were lethally irradiated without any shielding and transplanted with green fluorescent protein (GFP)-expressing bone marrow from DAGFP followed by immunization with MOG in adjuvant (Fig.6e).”

Reviewer #3 It appears that the DA Gsta4 group had a 3-fold higher mortality rate (5.8 vs 15.0) than the DA WT group. These data seem at odds with the statement that there was no difference in mortality between the groups. If this difference is real and significant, the authors should discuss this issue, or indicate otherwise.

**Reply:** This has been addressed.

***Change in text:*** *Results Page 12*

*“There were no differences in terms of disease incidence or day-of-onset between the groups (Fig.S4a), suggesting that the triggering of an autoimmune response did not differ between the strains.”*

***Change in text:*** *Discussion Page 15*

*“EAE in non-chimeric animals displayed a difference in mortality, since this was not present in the chimeric EAE set-ups, one cannot exclude Gsta4-dependent contributions from bone-marrow derived cells in non-chimeric EAE settings. “*

**Reviewer comments, second version:**

Reviewer #1 (Remarks to the Author):

The authors have addressed my primary concerns and have substantially improved their manuscript. I have no more major comments.

Minor comments:
1. Line 252, "DAwt axons were determined to be less myelinated after remyelination compared to DAGsta4." I think the investigators meant "after demyelination" rather than "after remyelination".

2. Figure 3 labeling seemed out of order "n, o, p, m" rather than "m, n, o, p"


Reviewer #3 (Remarks to the Author):

The PI has satisfactorily addressed the majority of the concerns that I had with this manuscript in its initial submission. The revised data clearly show that the fumarate moiety is the important biological effector rather than clemastine. Yet both the first and last paragraphs of the discussion highlight the clinical potential of Clem-F. This seems way out of place with respect to the main point of the paper which is the role of Gsta4 in myelin biology. That to me should be take away message from the discussion.

**Author rebuttal, second version:**

Reviewer #1 Line 252, "DAwt axons were determined to be less myelinated after remyelination compared to DAGsta4." I think the investigators meant "after demyelination" rather than "after remyelination".

**Reply:** This has been addressed.

***Change in text:***

*“Using a software that randomly selects axons for analysis from every image frame^59^, DA^Wt^ axons were determined to be less myelinated after demyelination compared to DA^Gsta4^ axons (Fig.5c, e, Supplementary Figure 3f).”*

Reviewer #1 Figure 3 labeling seemed out of order "n, o, p, m" rather than "m, n, o, p"

**Reply:** The labeling is not out of order.

Reviewer #3 (Remarks to the Author): The PI has satisfactorily addressed the majority of the concerns that I had with this manuscript in its initial submission. The revised data clearly show that the fumarate moiety is the important biological effector rather than clemastine. Yet both the first and last paragraphs of the discussion highlight the clinical potential of Clem-F. This seems way out of place with respect to the main point of the paper which is the role of Gsta4 in myelin biology. That to me should be take away message from the discussion.

**Reply:** This has been addressed.

***Change in text:***

“There has been some progress in the identification of signaling pathways involved in OL maturation. These include the Wnt pathway, interfering with remyelination in the mammalian CNS61 and retinoid acid receptor RXR-γ signaling that enhances OPC differentiation and remyelination62. A large scale high-throughput screening of compounds with remyelinating effects identified a cluster of anti-muscarinic formulations, including clemastine, that enhance OPC differentiation and remyelination in vitro63.”

***Change in text:***

“Facilitating and increasing remyelination has been a longstanding therapeutic goal in chronic demyelinating diseases such as MS67. Until very recently successful examples, especially in clinical settings, have been lacking. Our study places Gsta4 as a key regulator of OL differentiation and remyelination in the context of cellular stress, ageing and demyelination in an experimental setting.”

**Reviewer comments, third version:**

**Author rebuttal, third version:**
